# Evaluating the analgesic effect of metamizole in the mouse model for DSS-induced acute colitis

**DOI:** 10.3389/fphys.2026.1810607

**Published:** 2026-08-05

**Authors:** D. T. Jacob, L. M. Keubler, A. Glasenapp, K. Selke, M. Buettner, L. Wenzel, S. Buchheister, S. Lutscher, H. Bähre, M. Bankstahl, A. Bleich, C. Häger

**Affiliations:** 1 Institute for Laboratory Animal Science and Central Animal Facility, Hannover Medical School, Hannover, Germany; 2 Research Core Unit Metabolomics, Hannover Medical School, Hannover, Germany; 3 Institute of Pharmacology and Toxicology, Department of Biological Sciences and Pathobiology, University of Veterinary Medicine Vienna, Vienna, Austria

**Keywords:** 4-AA, 4-MAA, analgesia, IBD, refinement, severity assessment, wheel running, metamizole

## Abstract

A commonly used mouse model of ulcerative colitis is the Dextran Sodium Sulphate (DSS) colitis model, including symptoms of weight loss, softening of stool up to diarrhea, and abdominal pain. To address 3R-refinement, we aimed to reduce severity in this model through pain therapy with metamizole. In addition to the potential analgesic effect, we investigated whether metamizole had a modulatory effect on inflammatory processes. In the study, ten-week-old female C57BL/6J mice were used. For pharmacological analysis, mice were treated with 200 mg/kg/d metamizole over 2 days to calculate baseline plasma concentration of metamizole metabolites using LC-MS/MS. After 14 days, DSS was administered via drinking water for 5 days, followed by metamizole for 2 days to determine plasma concentration. To assess disease severity and pain therapy, mice received DSS via drinking water for 5 days, controls received water only. Mice were then treated with metamizole via the drinking water for an additional 5 days, while corresponding sub-cohorts were left untreated (water+water, water+metamizole, DSS+water, DSS+metamizole). For severity assessment, changes in bodyweight, posture, stool consistency, voluntary wheel running (VWR) and Mouse Grimace Scale (MGS) were analyzed. Furthermore, colon samples were used for histology and gene expression analyses. LC-MS/MS analysis revealed significantly higher plasma concentrations of 4-methylaminoantipyrine and increased concentrations of 4-aminoantipyrine following DSS and subsequent metamizole treatment when compared to baseline. Assessing disease severity in control mice (water+water or water+metamizole-treated) demonstrated no clinical signs or changes in VWR. DSS+water and DSS+metamizole treated mice showed similar loss of body weight and clinical signs. VWR performances were decreased in DSS+water-treated mice as well as in DSS+metamizole-treated mice. There were no statistically significant differences between untreated and metamizole-treated animals with DSS-colitis. Interestingly, none of the groups showed elevated MGS scores, nor did the gene expression analyses detect relevant differences. This study showed that administering metamizole via drinking water led to detectable levels of metamizole metabolites in the plasma of treated mice. However, metamizole did not reduce disease severity or pain in this mouse model. Therefore, other refinement strategies should be explored in future studies.

## 1 Introduction

*Colitis ulcerosa* and Crohn’s disease are widespread inflammatory bowel diseases with a prevalence of 300 to 500 cases per 100,000 people in Europe and North America (Ng et al., 2017). Due to their chronicity and episodic course, chronic inflammatory bowel diseases (IBD) cause a significant impairment to the quality of life and daily functioning in affected individuals. Current knowledge indicates that the pathogenesis of IBD is largely determined by the interaction of predisposing genetic and epigenetic factors, as well as the composition of the intestinal microbial flora. The fine regulation of pro- and anti-inflammatory mediators also plays a crucial role in the pathogenesis of the disease. It is assumed that an impaired immune regulation predisposes the occurrence of IBD (Willing et al., 2010; Keubler et al., 2015).

For experimental investigation of this disease, the dextran sulphate sodium (DSS) induced mouse model of acute colitis is frequently used, where DSS damages the intestinal epithelium and initiates an inflammatory reaction (Solomon et al., 2010). Furthermore, DSS causes dose-dependent softening of the stool up to diarrhea and weight loss, which are parameters used in clinical scoring to assess disease severity. Besides these, there are barely any clinical variables indicating disturbed well-being (Häger et al., 2015). In contrast, automated home-cage activity monitoring systems, particularly those including voluntary wheel running (VWR) monitoring, have been shown to be highly effective and sensitive for assessing compromised welfare in the DSS-colitis model (Häger et al., 2018; Weegh et al., 2020; Zentrich et al., 2021).

The burden in the DSS-colitis model is undoubtedly multidimensional, i.e., besides an impaired well-being, mice most likely experience abdominal pain (Eijkelkamp et al., 2007; López-Estévez et al., 2021; Vezza et al., 2023). This raises the question of the severity of the pain the animals experience and whether it can be treated without compromising the animal model.

Symptomatic pain is also part of the human disease and analgesics like non-steroidal anti-inflammatory drugs (NSAIDs) and opioids have been shown to modulate the underlying inflammatory reactions and pathomechanisms of IBD (Takeuchi et al., 2006; Coates et al., 2023; Grunkemeier et al., 2007).

The use of opioids or NSAIDs as refinement measures in IBD animal models is currently controversial (Golusda et al., 2022), and animals in these models have not routinely received analgesia. Data on the use of other analgesics with less anti-inflammatory activity, such as metamizole, are comparatively scarce (Spalinger et al., 2023).

The present project aimed to investigate whether treatment with a non-opioid analgesic in the acute DSS-colitis mouse model reduces the animals’ pain and whether this treatment influences the inflammatory response. The maximal intensity of colitis is typically reached two days after DSS withdrawal. At that time, the most pronounced histological and molecular changes as well as the greatest impairment of the intestinal barrier can be observed. Subsequent intestinal repair processes lead to gradual regeneration of the colon mucosa, so that inflammatory parameters gradually return to baseline. This recovery phase also plays a crucial role in human medicine, as it is essential to achieve a stable remission (Vidal-Lletjós et al., 2019).

Metamizole, administered via drinking water, is the chosen drug for analgesia in this study. Besides its analgesic, antipyretic, and antispasmodic properties, metamizole has shown to have a lower impact on peripheral inflammatory processes than NSAIDs. For example, a study showed that the oral administration of metamizole reduced abdominal pain in a murine model of acute pancreatitis without affecting important model-specific parameters (Stumpf et al., 2016).

While selecting the analgesic for this study, besides potential side-effects, we also took the duration of action and the associated route and frequency of administration into account. Frequent handling and restraint of animals for subcutaneous or intraperitoneal injections are known to cause additional stress and should be avoided when possible (Balcombe et al., 2004; Meijer et al., 2006). Hence, a stress-free method of analgesic administration via drinking water was planned as a potential refinement in this study. Due to the experimental design, analgesic administration can only commence after discontinuing the administration of DSS via drinking water on day 5. Since, as mentioned earlier, the manifestation of colitis symptoms is expected to peak on day 7 (two days after stopping DSS administration), the treatment with the analgesic on day 5 provided sufficient time to evaluate its effect on colitis-induced symptoms.

To examine the burden on the animals with a focus on pain recognition, the mice were closely monitored using a multivariate approach including the automated monitoring of voluntary wheel running in the home cage, the Mouse Grimace Scale (MGS) and a model-specific clinical score.

## 2 Materials and methods

### 2.1 Ethical statement

All procedures in this study were conducted with the approval of the Lower Saxony State Office for Consumer Protection and Food Safety (LAVES, license Nr. 33.12-42502-04-22-00239), in accordance with the European Directive 2010/63/EU and the German law for animal protection. The study was preregistered in AnimalStudyRegistry.org: DOI 10.17590/asr.0000309.

### 2.2 Animals and housing conditions

In the study adult, female C57BL/6J mice bred in the central animal facility of the Hannover Medical School were used. The mice were between 10 to 13 weeks of age at the start of the experiments. For the duration of the study, the mice were housed in an experimental room designated for behavioral studies, and environmental parameters were maintained at a constant temperature (22 °C ± 2 °C), humidity (55% ± 10%) and light cycle (14:10-hour light/dark cycle). The mice were fed ad libitum with standard pelleted rodent feed (Altromin 1324 TPF Maintenance Diet, Lage, Germany) and had free access to autoclaved drinking water. Animal cages were lined with poplar wood granulate bedding (Thomsen DeLuxe Bedding “LAB.BED”; poplar wood AB 3–180 V, Handewitt, Germany) and were enriched with a cotton roll as nesting material as well as a tunnel (Mouse Tunnel, red, Plexx BV, Netherlands). Other nesting material was omitted to prevent any hindrance to the running wheel activity of the mice. The same female experimenters conducted most procedures and caretaking during the light phase between 8AM and 12PM. For all husbandry purposes and experimental procedures, the mice were either tunnel- or cup-handled. Routine microbiological monitoring according to FELASA recommendations did not reveal any evidence of infection with common murine pathogens (Mähler et al., 2014).

### 2.3 Study design part A: pharmacological analysis of metamizole intake

**Figure 1:**
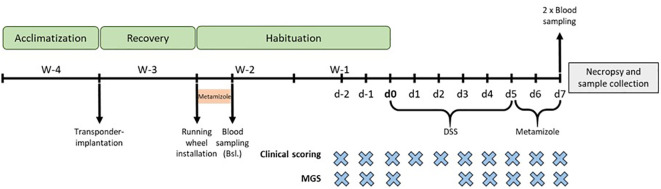
Timeline of pharmacokinetic experiments. (MGS, Mouse Grimace scale; Bsl, baseline; DSS, Dextran sulphate sodium).

A week after being brought to the experimental room, the mice (n=16) were implanted with RFID transponders in the sub-cervical region, to track their individual running wheel activities. Following a week of recovery, all mice were administered 200mg/kg/d of metamizole (Novaminsulfon 500mg Lichtenstein, Zentiva Pharma GmbH, Frankfurt am Main) over two days (d-14 and d-13) to calculate the baseline ad libitum intake of metamizole (controls). At the same time, the running wheels were also installed to allow the mice to acclimatize to them and achieve a stable running rate. At the end of the 48-hour metamizole administration, blood samples were taken (d-12). After two weeks of recovery, we began administration of 2% DSS via drinking water over five days (d0-d5). This was followed by a 48-hour oral metamizole treatment via drinking water (d5-d7). Blood samples were taken at a twelve-hour interval, which was 36 hours (active phase) and 48 hours after beginning metamizole administration. The mice were humanely killed by CO_2_ inhalation in a closed cage with a 25% flow rate of chamber volume per minute followed by cardiac puncture and exsanguination on day 7, and samples were collected (**Figure 1**).

#### 2.3.1 Metamizole administration, blood sample collection and LC-MS/MS analysis

Metamizole (Novaminsulfon 500mg Lichtenstein, Zentiva Pharma GmbH, Frankfurt am Main) was freshly prepared with filtered tap water daily and given to the mice in darkened water bottles (Tecniplast, Italy). The concentration of metamizole in the drinking water was individual to each group and determined by taking the desired dosage (see above), average baseline water consumption measured over three days prior to DSS administration (d-2 to d0) and mean body weight of the mice in the group into consideration. For the blood sampling procedure, the mice were fixated in a custom-built restrainer. Approximately 120µl of blood was drawn from the lateral tail vein of the mice by scalpel micro-incision. For a detailed description of the blood withdrawal technique and LC-MS/MS analysis refer to the publication by Glasenapp and colleagues (Glasenapp et al., 2024).

#### 2.3.2 Clinical score

During the experimental procedures, we clinically scored the mice daily to assess their state of well-being. This included the monitoring of parameters such as activity, loss of body weight, general appearance, stool consistency, and wound healing following the transponder-implantation. Humane endpoints were also defined in the score sheets. Based on scores of individual parameters as well as the total score (**Supplementary Table 1**), in Part A, 1 out of 16 mice had to be euthanized on day 6 due to a decline in bodyweight by >20%, reduced activity and changes in the general appearance.

### 2.4 Study design part B: analysis of VWR and MGS

**Figure 2:**
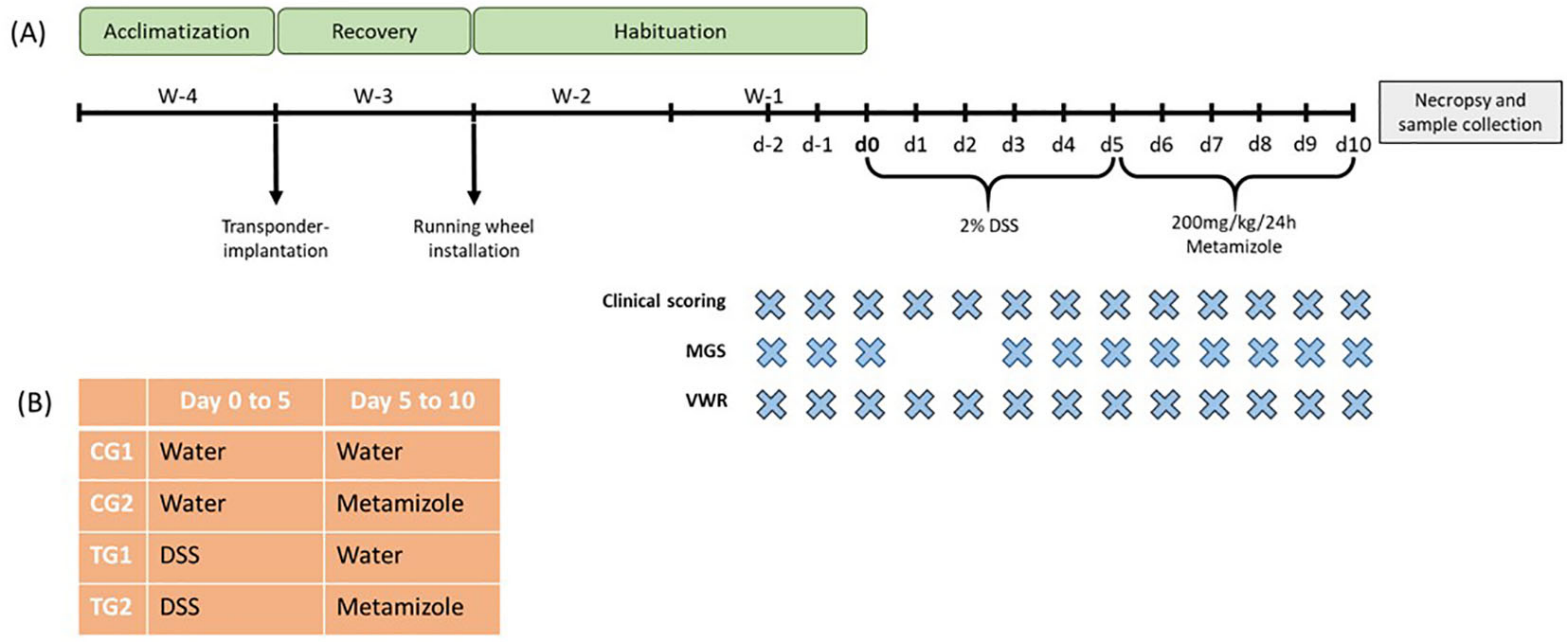
**(A)** Timeline of the behavioral analysis experiments. **(B)** Classification of the experimental groups and treatment received. (CG, Control Group; TG, Test Group; DSS, Dextran sulphate sodium; MGS, mouse grimace scale).

For Part B, a new group of mice (n=64) were brought to the experimental room four weeks prior to the administration of DSS. Following an acclimatization period of one week, we implanted the mice with RFID transponders. The mice had a week to recover from the surgery, after which the running wheels were installed in the cages. This was followed by a two-week adaptation period for the mice to habituate themselves to the running wheels and achieve a stable running rate. We recorded the baseline values for the clinical score, mouse grimace scale, voluntary wheel running, and drinking water intake over three days prior to the application of DSS (d-2 to d0). For this study, we analyzed four treatment groups. The first group consisted of mice that received water only (control group 1, CG1). The second group received water from day 0 to day 5, followed by metamizole treatment from day 5 to day 10 (control group 2, CG2). The third group was treated with DSS for five days (day 0 to day 5) and then received water from day 5 to day 10 (treatment group 1, TG1). The fourth group received DSS from day 0 to day 5 and was additionally treated with metamizole from day 5 to day 10 (treatment group 2, TG2). On the tenth day, we humanely killed the mice by CO_2_ inhalation in a closed cage with a 25% flow rate of chamber volume per minute followed by cardiac puncture and exsanguination and collected samples for the histological analysis (**Figure 2**). Due to a decline in bodyweight by >20%, reduced activity and changes in the general appearance, 5 out of 16 mice in TG1 and 6 out of 16 mice in TG2 had to be euthanized between days 8 and 10.

#### 2.4.1 Wheel running analysis

We used the IDRevolyzer running wheel system (PhenoSys GmbH Berlin, Germany and preclinics GmbH Potsdam, Germany), to house the mice and track their running activity. Twelve EU Type II Macrolon^®^ cages were used to house 16 mice at a time. Every three adjacent cages housed a group of four mice and were joined to each other using plastic tubes, thus allowing the mice free access to all three cages. The cages were equipped with running wheels that were attached to the system through a hole at the back of the cage. Behind the wheel axel a radio-frequency identification (RFID) receiver was installed to identify individual mice using subcutaneously implanted RFID transponders, thus recording individual wheel running activity.

For transponder implantation into the sub-cervical region, we induced anesthesia using 4–5 vol% isoflurane (Isofluran CP^®^ 1 ml/ml, CP-Pharma Handelsges. mbH, Burgdorf, Germany) and maintained it at approximately 1–2 vol% isoflurane. Absence of the righting- and withdrawal reflex confirmed sufficient depth of anesthesia. Mice were placed on a warming pad in a dorsoventral position and eye ointment (Bepanthen^®^, Bayer Vital GmbH, Leverkusen, Germany) was applied to protect the cornea. To prepare the surgical site, we clipped the fur in the region between the center of the neck and approximately 1cm caudal to the shoulder blades. After disinfecting the skin with an alcohol-based disinfectant (Softasept^®^ N, B. Braun Melsungen AG, Melsungen, Germany), a local anesthetic cream (Emla^®^ 25mg/g + 25mg/g Crème lidocaine/prilocaine, Aspen Pharma Trading Limited, Dublin, Ireland) was applied to the surgical site. After implanting the transponder subcutaneously, we closed the opening with two interrupted sutures (Vicryl Rapide™ 5-0, Ethicon^®^ Johnson & Johnson Medical GmbH, Norderstedt, Germany) and a small amount of tissue adhesive (Surgibond, SMI AG, St. Vith, Belgium). We carefully monitored the mice during the entire procedure and clinically scored them in the days following surgery. The software Phenosoft Control (PhenoSys GmbH Berlin, Germany) generated the voluntary wheel running (VWR) activity data, and this data was imported using the WheelAnalytics (version 1.0.0) software (Details on study design: **Figure 2**).

#### 2.4.2 Mouse grimace scale

We collected the footage to analyze the MGS by placing the mice in observation boxes (9x5x5 cm³) made of Plexiglas. The boxes were placed inside an illuminated photo tent (Helios photo light tent 80 x 80 cm Luxburg^®^ Studioset, Luxburg GmbH, Gelsenkirchen, Germany) and we recorded the mice for approximately 10 to 12 minutes using a Canon EOS 250D camera (Canon Deutschland GmbH, Krefeld, Germany). To acclimatize the mice to the observation boxes, we trained them using a protocol (**Supplementary Table 2**) for three days prior to the baseline recordings. During the training, the mice were placed in the observation boxes for increasing durations of time and on the final day of training, the boxes were placed in the photo light tent for the entire duration of the recording. For the baseline, videos were recorded on three days prior to the administration of DSS. Following this, footage was collected from the third day of the DSS application until the end of the experiment (d3 to d10). After the experiment, two people blinded to the proceedings of the experiment prepared the footage for evaluation. They selected five representative pictures of each mouse from different time points in each video. These photos were shuffled and presented to two experienced scorers blinded to the experimental setup who then used a semi-automated software (Ernst et al., 2020) to score the facial action units (FAU) of the mice. The results generated by the software were assigned to the respective mouse and day of the experiment and the average of the five photos as well as the two scorers were taken into account while assigning the final scores to the individual mice (For details on study design see **Figure 2**).

#### 2.4.3 Drinking water uptake

The total intake of drinking water of all the experimental groups was monitored during DSS and metamizole administration. We measured the intake of drinking water in the group-housed mice by weighing the water bottles. Three bottles were shared between four mice, and the total intake of the group was calculated and compared to baseline values taken over three days (d-2 to d0). To account for the water lost while removing and placing the bottles back in the cage, the contents of all the water bottles (test and control groups) were kept the same.

#### 2.4.4 Histology

The mice were humanely euthanized via CO_2_ inhalation followed by cardiac puncture and exsanguination on the last day of the experiment (d7/d10) after collecting the final blood samples. After separating the colon from the rest of the gastrointestinal tract and flushing it with phosphate-buffered saline (PBS), it was placed in a biopsy cassette using the swiss-roll technique without opening the colon (Moolenbeek and Ruitenberg, 1981). The cassette was immediately immersed in formalin (Biochrom GmbH, Berlin) and later embedded in paraffin wax. Slices of the colon embedded in the paraffin were stained using hematoxylin and eosin (H&E) and analyzed further. For the microscopic analysis, an experienced scorer blinded to the experimental setup assessed the proximal and distal colon and scored them separately according to a previously published histological score (Häger et al., 2015) (see also **Supplementary Table 3**).

### 2.5 Study design part C: molecular biological analysis

**Figure 3:**
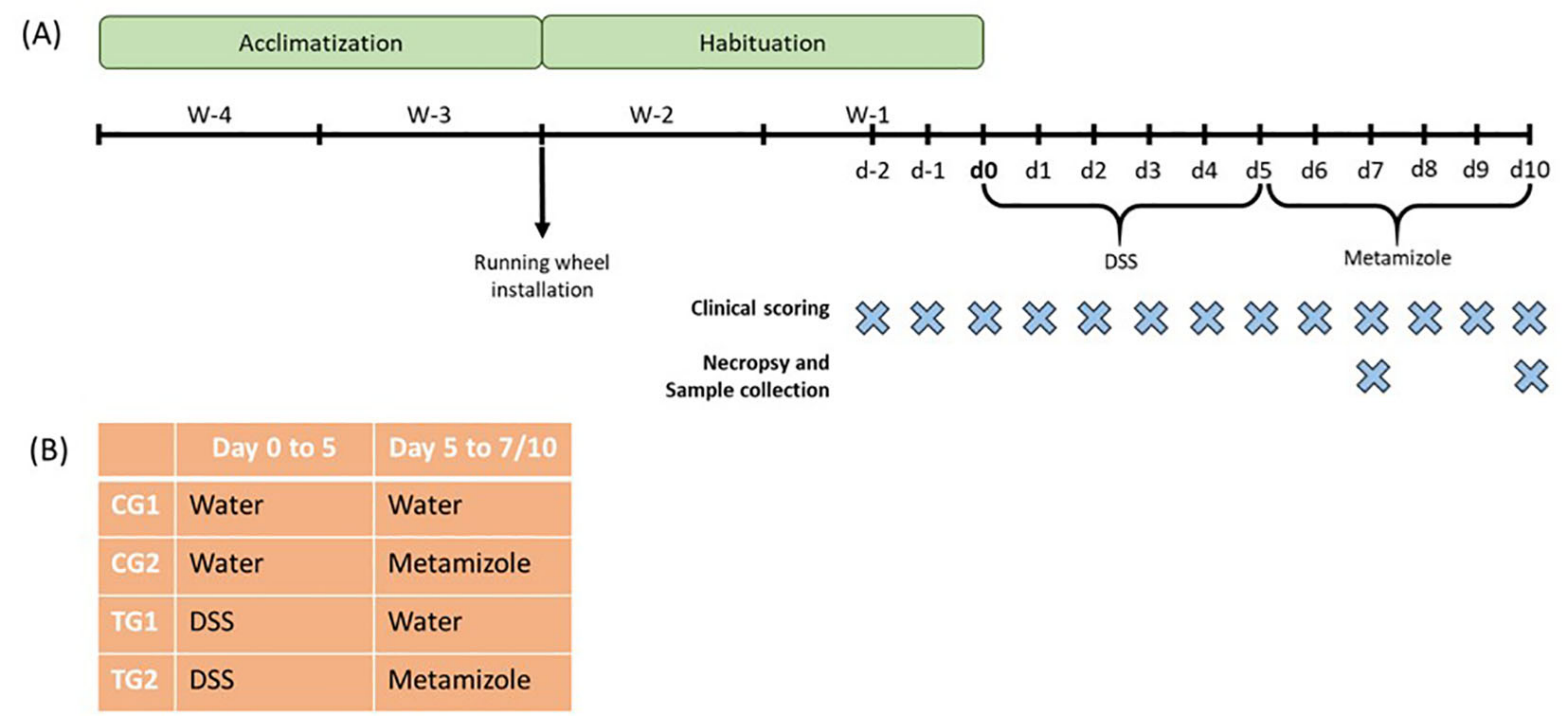
**(A)** Timeline of the molecular biological analysis experiments. **(B)** Classification of the experimental groups and treatment received. (CG, Control Group; TG, Test Group; DSS, Dextran sulphate sodium).

Mice (n=80; including 32 from Part B) had an acclimatization period of two weeks to the experimental room before we installed the running wheels in the cages. Following a two- week habituation period to the running wheels, we began with DSS application followed by oral administration of metamizole via drinking water. For gene expression analyses mice were humanely killed by CO_2_ inhalation in a closed cage with a 25% flow rate of chamber volume per minute followed by cardiac puncture and exsanguination on d7 and on d10 to analyze the extent of intestinal inflammation at these timepoints (**Figure 3**).

#### 2.5.1 RNA extraction from colon samples

To isolate RNA from colon tissue, an ~0.2 cm piece of tissue was extracted from the distal colon and transferred in an Eppendorf tube containing 600 µl of RLT-buffer (QIAGEN GmbH, QIAGEN Strasse 1, 40724 Hilden, Germany) and Dithiothreitol (DTT) mixture along with a stainless-steel bead. The tissue was disrupted and homogenized using a TissueLyser (TissueLyserII, QIAGEN GmbH), at a frequency of 30 Hz for two minutes. The lysate was either frozen at -80°C or directly processed to extract the RNA according to the protocol provided by the RNeasy Mini Kit (QIAGEN GmbH). RNA concentration was measured using a spectrophotometer (NanoDrop^®^ ND-1000) and the RNA was stored at -80 °C for further analysis.

#### 2.5.2 qRT-PCR

The cDNA was synthesized from the isolated RNA using the QuantiTect^®^ Reverse Transcription Kit (200) (QIAGEN GmbH). To eliminate contaminating gDNA, RNA samples were incubated in a Wipeout Buffer at 42 °C for approximately 2 minutes. For the reverse transcription, a master mix containing Quantiscript Reverse Transcriptase, Quantiscript RT Buffer and RT Primer Mix was added to the samples and incubated at 42 °C for 15 min. The samples were incubated at 95 °C for 3 min to inactivate the Quantiscript Reverse Transcriptase. The cDNA was diluted with RNAase free water (QIAGEN GmbH) for further analysis. During the synthesis, the RNA samples and contents of the kit were placed on ice.

For the quantitative real-time PCR analyses, TaqMan^®^ (applied Biosystems^®^, life technologies™) and the Sybr^®^ Green (applied Biosystems^®^, life technologies™) chemistries were used with the QuantStudio^®^ 6 Flex Real-Time PCR System (Applied Biosystems^®^). Fast 384-Well Plates (MicroAmp^®^ Optical 384-Well Reaction Plate with Barcode, applied biosystems^®^, life technologies™) were used for the qPCR runs and Eef2 was selected as the housekeeping gene. All analyses were carried out on the Thermo Fischer Connect™ cloud-based software using the comparative Ct (ΔΔCt) method to calculate the relative gene expression (For details on the Primer list, reaction mix and thermal protocols refer to **Supplementary Tables 4**-**10**).

### 2.6 Statistics

Animal numbers were calculated using G*Power 3.1 Software for part A-C separately, according to the respective analysis. All statistical analyses were conducted using GraphPad Prism^®^ (v9.5.0.730, GraphPad Software, San Diego, California USA). Data was tested for normal distribution using the Shapiro-Wilk test and by analyzing the generated QQ plots. Values are presented as mean ± standard deviation (SD) unless indicated otherwise. The significance level was set at alpha= 0.05 and was summarized in the graphs as *p≤ 0.05, ** p≤ 0.01, *** p≤ 0.001 and **** p≤ 0.0001 (* was replaced by other symbols in some graphs).

For the change in bodyweight, voluntary wheel running data and drinking water intake during DSS and metamizole administration, the four groups were compared to each other using a mixed effects model followed by a Tukey’s multiple comparison test since the data included missing values. Normally distributed data within the groups were compared to values on day 0 by using either a mixed effects model followed by a Tukey’s multiple comparison test or for data without missing values, a RM one way ANOVA followed by a Dunnett’s multiple comparison test. The colon histology scores were analyzed using the Kruskal-Wallis test followed by a Dunn’s multiple comparison to compare the groups with each other. The values in this graph are plotted as median with an interquartile range (IQR). For the clinical score and MGS, the groups were analyzed together using the Mann-Whitney test with the Holm-Šídák method. Within the groups, the change in scores over the days were compared to day 0 using the Wilcoxon signed-rank test (hypothetical value “0”). The MGS values are plotted as Median with a 95% confidence interval (CI).

In the gene expression analyses, the four groups were analyzed using the two-way ANOVA followed by a Tukey’s multiple comparison for normally distributed data or a Kruskal-Wallis test followed by a Dunn’s multiple comparison. Outliers in the gene expression data were excluded using Rout’s method.

## 3 Results

### 3.1 Part A: intake of metamizole-medicated water and plasma concentrations of metamizole metabolites in the DSS colitis mouse model

To investigate how much metamizole was consumed by the mice the total intake of drinking water was measured. Additionally, the concentration of metamizole metabolites in the plasma was determined by conducting an LC-MS/MS analysis. To monitor the well-being of the mice, a daily clinical evaluation including the change in bodyweight, general appearance, activity, and stool consistency was conducted. Due to meeting the humane endpoint criteria, one mouse had to be euthanized on d6.

#### 3.1.1 Plasma concentrations of metabolites 4-MAA and 4-AA

**Figure 4:**
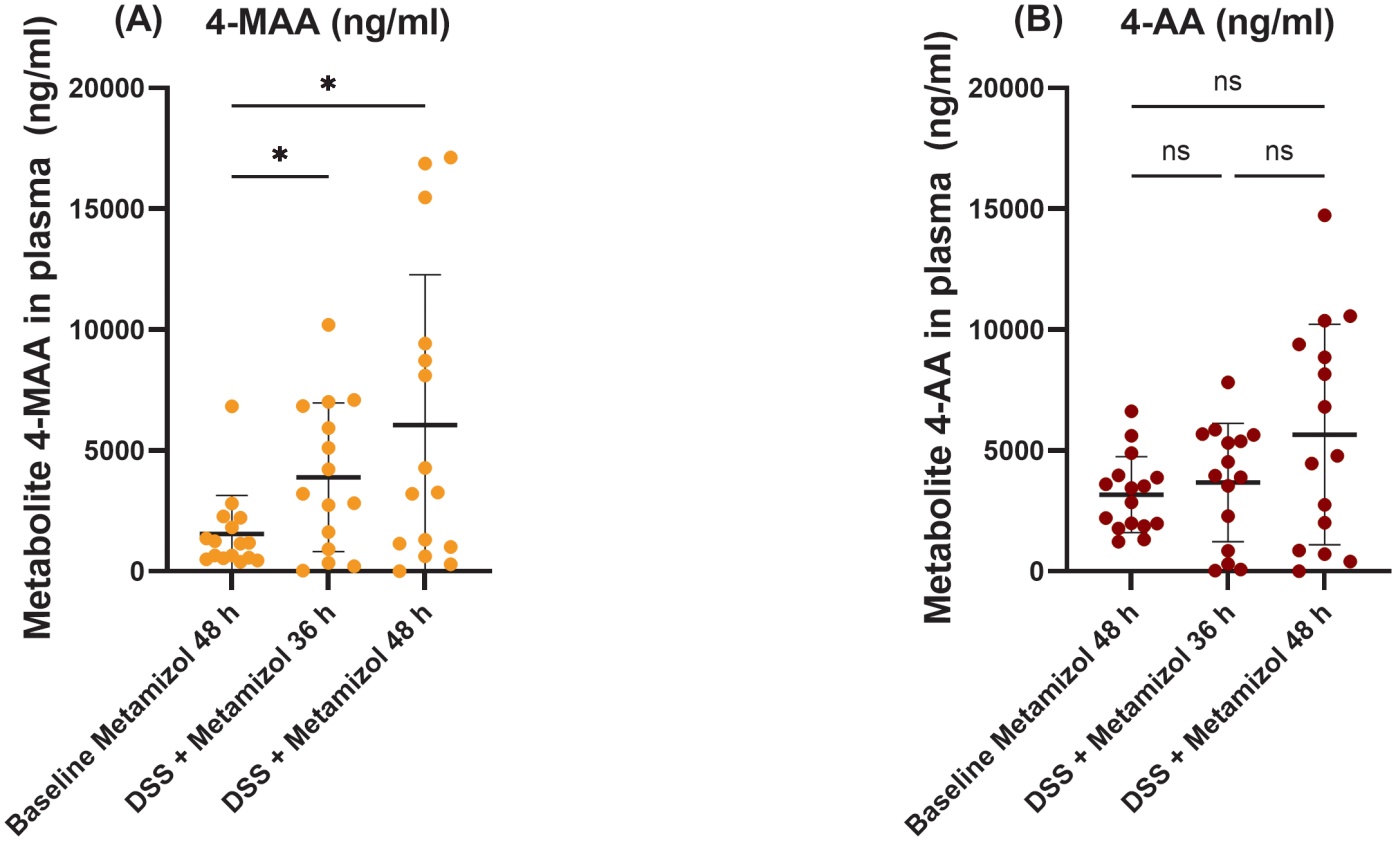
Plasma concentration of metamizole metabolites 4-methylaminoantipyrine (4-MAA) and 4-aminoantipyrine (4-AA). The concentrations of 4-MAA **(A)** and 4-AA **(B)** at various time points during the experiment was determined using liquid chromatography mass spectrometry. The analysis was conducted using a linear mixed effects model fixed effect type III with a Tukeys’s multiple comparisons test (p =<0.05).; **(A)** Fixed effect type III: p value 0.0176, F (1.376, 19.27) = 5.860; **(B)** Fixed effect type III: p value 0.0765; F (1.541, 21.58) = 3.104). (n=16: Baseline Metamizole 48h; n=15: DSS+Metamizole 36h and 48h). P was considered significant at ≤ 0.05 with * as p≤ 0.05; ** as p≤ 0.01; *** as p≤ 0.001; **** as p≤ 0.0001.

Plasma concentrations of the two active metamizole metabolites 4-MAA (**Figure 4A**) and 4-AA (**Figure 4B**) were analyzed using LC-MS/MS. The baseline values were measured two weeks before DSS administration and revealed a mean concentration of 1538 ng/ml (*SD* = 1595) of the 4-MAA metabolite, while the mean concentration of 4-AA was at 3172 ng/ml (*SD* = 1573). After the five-day DSS administration, a two-day treatment with metamizole was started, and blood samples were collected 36 hours and 48 hours after starting the metamizole treatment. The 4-MAA metabolite concentration of 3886 ng/ml (*SD* = 3074) 36 hours after starting metamizole administration was significantly higher (p = 0.024) when compared to baseline. Twelve hours later, the concentration of 4-MAA in the plasma increased further to 6057 ng/ml (*SD* = 6220) and was also significantly higher than the baseline concentration (p = 0.018). Although LC-MS/MS analysis demonstrated an elevated mean 4-AA concentration of 3676 ng/ml (*SD* = 2448) 36 hours after starting metamizole treatment as well as at 48 hours with 5658 ng/ml (*SD* = 4561), this was not statistically significant when compared to baseline.

#### 3.1.2 Clinical and histological evaluation

The daily clinical evaluation included an assessment of the change in bodyweight. On comparing the relative change in bodyweight over the seven days to values on d0, the data showed a significant increase over the first three days of DSS administration with the highest value observed on d2 (p = 0.0012). This was followed by a significant decline from d4 onwards and continued during metamizole administration (d5 - 7). The maximum decline in bodyweight was observed on day 7 with a reduction of 9.73% (*SD* = 7.21) (**Supplementary Figure 1**). These results were also reflected in the clinical score (**Supplementary Figure 2A**), where a significant increase in the total score was observed beginning from d3 and maximum values were seen on d7 with a score of 3.2 (*SD* = 2.95) out of a possible 15. Following the necropsy, a histological evaluation (**Supplementary Figure 2B**) confirmed colonic inflammation through the changes in the colon morphology and infiltration of immune cells. These changes contributed to a mean histological score of 19.07 (*SD* = 6.3).

### 3.2 Part B: analysis of the analgesic effect of metamizole during DSS-induced colitis using VWR and MGS

For this part of the study, four groups were analyzed during the DSS and subsequent metamizole administration: two control groups receiving water instead of DSS or metamizole (CG1, CG2) and two treatment groups receiving DSS with or without metamizole treatment (TG1, TG2). For the assessment of the analgesic effect of metamizole on the pain induced by DSS colitis, the two robust parameters of severity and pain assessment VWR and MGS were used. Additionally, daily clinical evaluation, analysis of the drinking water intake, and histological analysis were carried out. A total of 11 out of 64 mice had to be euthanized prior to the end of the experiments due to reaching their humane endpoint criteria (see also **Supplementary Table 1**).

#### 3.2.1 VWR analysis

To investigate whether the administration of metamizole reduced pain due to DSS-induced colitis, the daily individual voluntary wheel running activity of the mice was recorded.

**Figure 5:**
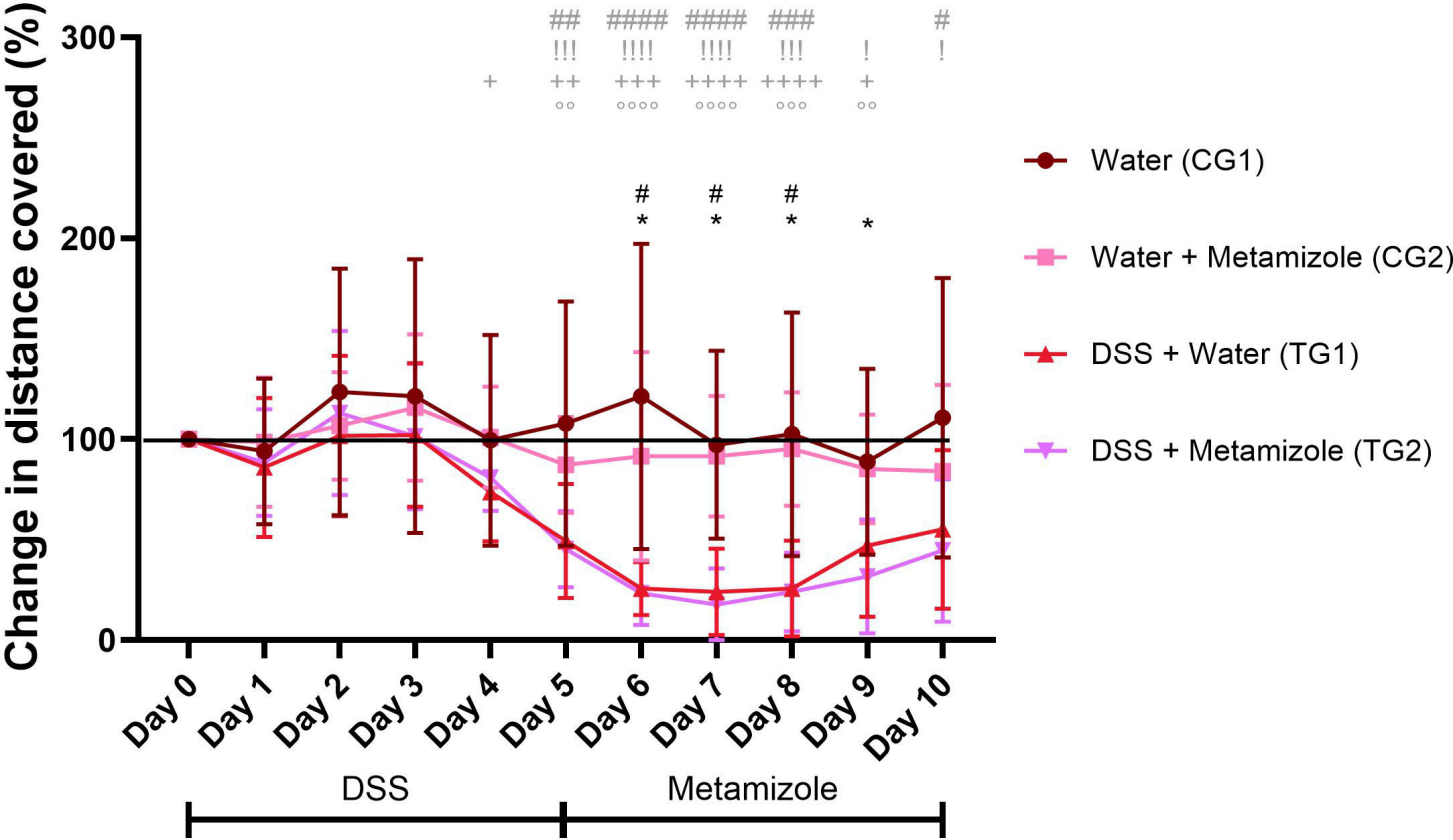
Change in the distance covered during voluntary wheel running on DSS and metamizole application. Change in the total distance covered over 20 hours (12 pm – 8 am) compared to the Day 0 (100%). The groups Water (CG1) vs. Water+Metamizole (CG2) (§-§§§); Water (CG1) vs. DSS + Water (TG1) (#-###); Water (CG1) vs. DSS + Metamizole (TG2) (!-)!!!; Water+Metamizole (CG2) vs. DSS + Water (TG1) (+-+++); Water+Metamizole (CG2) vs. DSS + Metamizole (TG2) (°-°°°); DSS + Water (TG1) vs. DSS + Metamizole (TG2) (*-***) were compared to each other using a Kruskal-Wallis test followed by a Dunn’s multiple comparison (grey symbols). Within the groups, the change in distance covered was compared to the baseline using the Friedman test followed by Dunn’s multiple comparison (CG1 (§) and CG2 (°), or a Kruskal-Wallis test with a Dunn’s multiple comparison (TG1 (#) and TG2 (*)). Only a significance of p<0.0001 in the in-group analysis is shown on the graph. The n= 15 in TG2 and n=16 in all other groups until day 7, n=14 on day 8, n=12 on day 9 and n=11 on day 10 in TG1 (DSS+Water); n=11 on day 8, n= 9 on day 9 and n=9 on day 10 in TG2 (DSS+Metamizole). P was considered significant at ≤ 0.05 with * as p≤ 0.05; ** as p≤ 0.01; *** as p≤ 0.001; **** as p≤ 0.0001.

On comparing the total distance covered by the mice in CG1 and TG1, significant differences were detected from d5 to 9 with a maximum reduction of 75.94% (*SD* = 25.45) observed on day 7 (p = <0.0001). The analysis also revealed significant differences between CG2 and TG2 on d5 to 9 and a significant reduction of 82.33% (*SD* = 18.04) in the total distance covered by the TG2 group was detected on day 7 (p = <0.0001; **Figure 5**). Both test groups displayed a gradual increase in VWR from d8 onwards and on the final day of the experiment (d10), the total distance covered by the mice in the TG1 was 44.83% (*SD* = 39.44) below baseline values. Similarly, in the mice that were treated with metamizole following DSS administration (TG2), the total distance covered was 55.33% (*SD* = 35.5) below the baseline (**Figure 5**).

**Figure 6:**
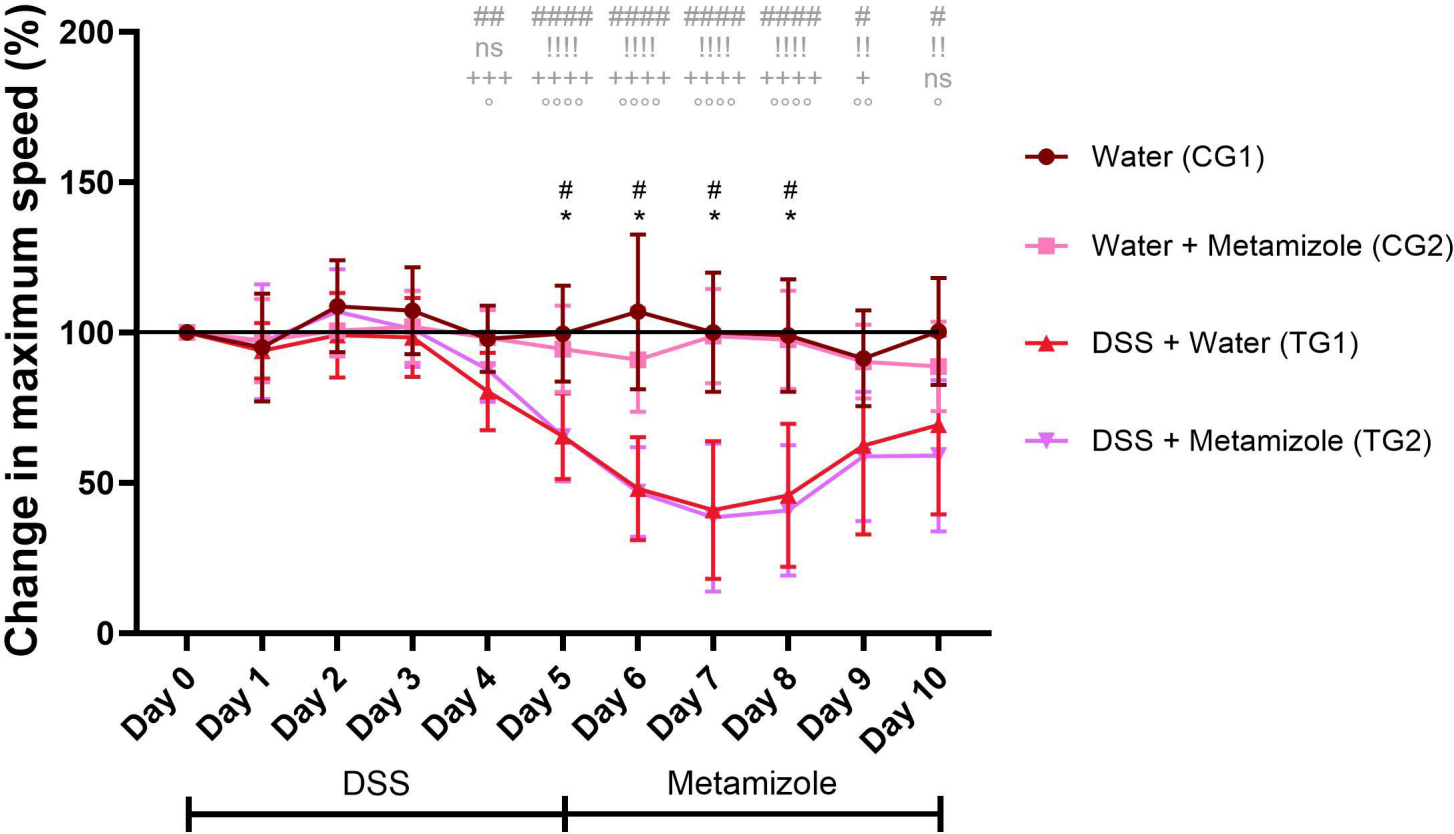
Change in the maximum speed achieved in voluntary wheel running during DSS and metamizole application. Change in the sum of the maximum speed achieved over 20 hours (12 pm – 8 am) compared to Day 0 (100%). The groups Water (CG1) vs. Water+Metamizole (CG2) (§-§§§); Water (CG1) vs. DSS + Water (TG1) (#-###); Water (CG1) vs. DSS + Metamizole (TG2) (!-)!!!; Water+Metamizole (CG2) vs. DSS + Water (TG1) (+-+++); Water+Metamizole (CG2) vs. DSS + Metamizole (TG2) (°-°°°); DSS + Water (TG1) vs. DSS + Metamizole (TG2) (*-***) were compared to each other using a Mixed-effects model (REML), Fixed effects (type III) followed by a Tukey’s multiple comparison test (grey symbols). Within the groups, the sum of the maximum speed was compared to the baseline using a Mixed-effects model (REML), Fixed effects (type III) followed by a Dunnett’s multiple comparison test (CG1 (§), CG2 (°), TG1 (#) and TG2 (*)). Only a significance of p<0.0001 in the in-group analysis is shown on the graph. The n= 15 in TG2 and n=16 in all other groups until day 7, n=14 on day 8, n=12 on day 9 and n=11 on day 10 in TG1 (DSS+Water); n=11 on day 8, n=9 on day 9 and n=9 on day 10 in TG2 (DSS+Metamizole). P was considered significant at ≤ 0.05 with * as p≤ 0.05; ** as p≤ 0.01; *** as p≤ 0.001; **** as p≤ 0.0001.

In addition to analyzing the total distance covered during wheel running activity, the maximum speed achieved by the mice on the running wheel was also monitored (**Figure 6**). The comparison between CG1 and TG1 as well as the CG2 and TG2 groups showed significant differences between d4 and 10 with the maximum effect observed on d7 in both groups (p = <0.0001). Beginning at d4, a significant reduction in the maximum speed was observed in both test groups until d7. The TG1 showed a maximum reduction of 59.06% (*SD* = 22.86) on d7 followed by an increase to 30.75% (*SD* = 29.78) of the baseline values on d10. Similarly, following a maximum reduction of 61.53% (*SD* = 24.64) in TG2 on d7, an increase to just 41% (*SD* = 25.19) of the baseline values was documented at the end of the experiment (d10) (**Figure 6**).

There were no significant differences in the VWR activity between the TG1 and TG2. However, at the end of the experiment the total distance covered, and maximum speed were still below baseline levels in contrast to the bodyweight that was back to baseline values on d10.

#### 3.2.2 MGS

**Figure 7:**
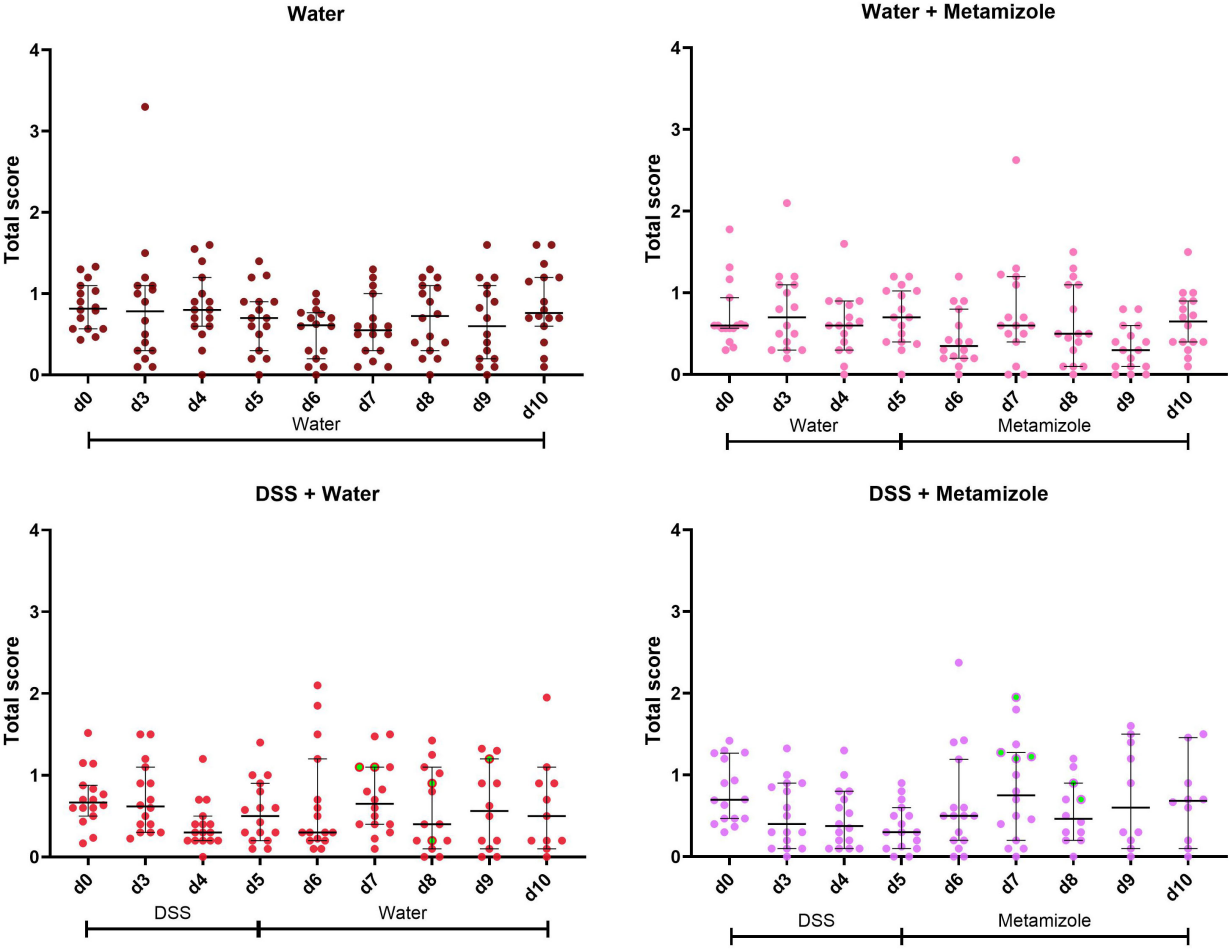
Total MGS score during DSS and metamizole administration. The groups were compared to each other using a Mann-Whitney-Test with the Holm-Šídák method. Within the groups, the change in the score was compared to Day 0 using the Wilcoxon signed-rank test to com compare the score values with the baseline (hypothetical value of ‘0’. Only a significance of p<0.0001 within the in-group analysis is shown on the graph. The n= 16 in each group till day 7, n=14 on day 8, n=12 on day 9 and n=11 on day 10 in TG1; n=12 on day 8, n=10 on day 9 and n=10 on day 10 in TG2. DSS administration: d0-d5 in TG1 and TG2; Metamizole administration: d5-d10 in CG2 and TG2; The scores of the animals that were euthanized due to reaching the humane endpoint before the end of the experiment (TG1 and TG2 Days 9 and 7) are displayed with different colors on the graph. Values are plotted as Median with 95% CI. P was considered significant at ≤ 0.05 with * as p≤ 0.05; ** as p≤ 0.01; *** as p≤ 0.001; **** as p≤ 0.0001.

To further analyze the analgesic effect of metamizole on the mice that received DSS, MGS was analyzed as a well described indicator of pain in mice. However, the analyses only revealed a significant difference in the total MGS score between CG1 and TG1 on d3 (p = 0.000812), where the control group showed higher scores. There were no other significant differences between the control and test groups (**Figure 7**). Within each experiment group as well, we did not find any significant changes in the MGS compared to baseline. Out of all the FAUs, highest scores were seen in the orbital tightening (maximum 1.4) and ear position (maximum 1.07) (**Supplementary Figures 3**, **4**). The mice that were euthanized because of meeting the humane endpoint criteria showed total MGS scores between 0.7 and 1.9 (highlighted in **Figure 7**). Graphical illustrations of the remaining FAUs (Nose bulge and Whisker Change) are provided with the supplementary material (**Supplementary Figures 5**, **6**).

#### 3.2.3 Clinical evaluation of DSS-colitis

In addition to the VWR and MGS analysis, a daily clinical evaluation was conducted during the DSS and metamizole administration (**Supplementary Table 1**).

**Figure 8:**
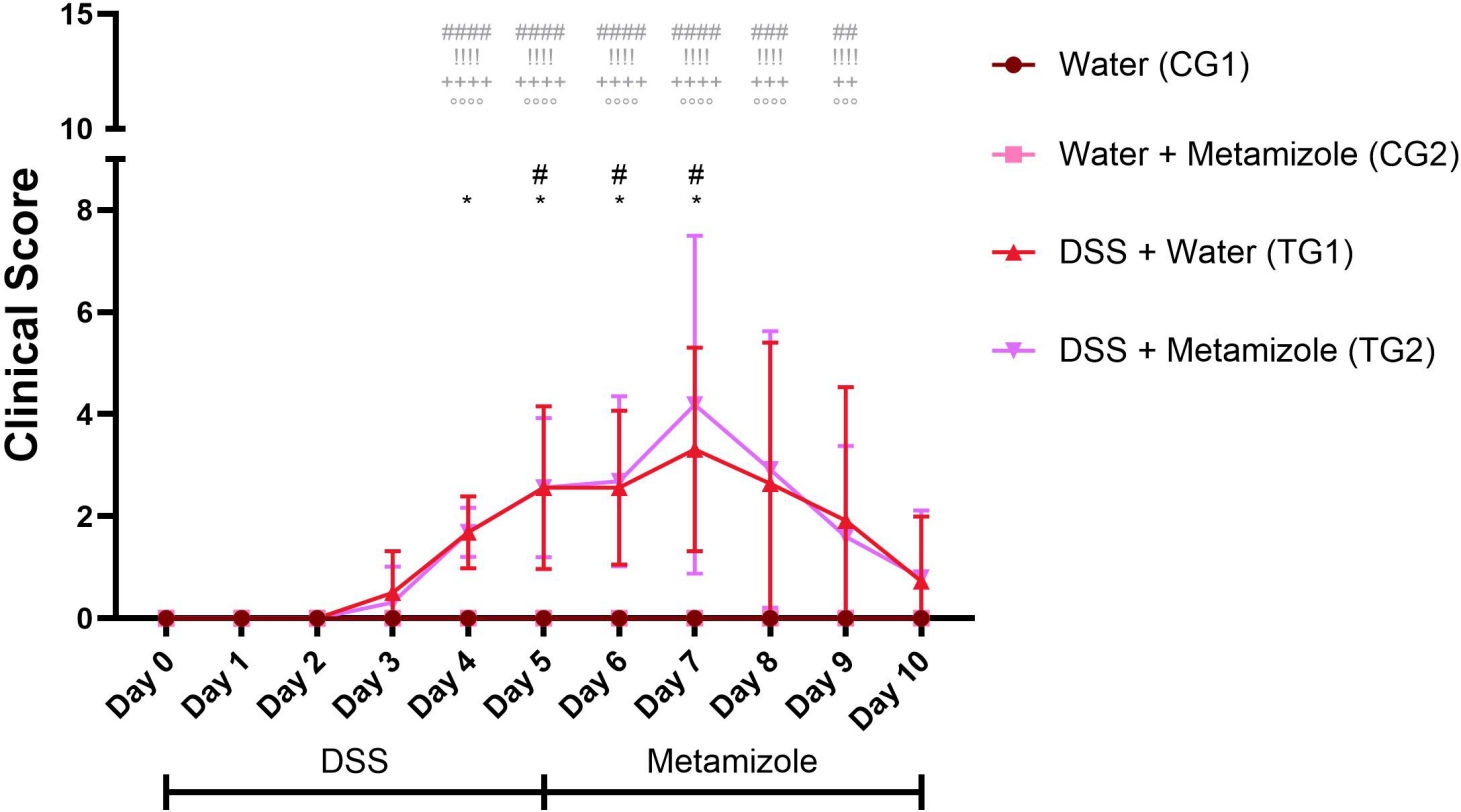
Clinical score during DSS and metamizole application. The groups Water (CG1) vs. Water+Metamizole (CG2) (§-§§§§); Water (CG1) vs. DSS + Water (TG1) (#-####); Water (CG1) vs. DSS + Metamizole (TG2) (!-)!!!!; Water+Metamizole (CG2) vs. DSS + Water (TG1) (+-++++); Water+Metamizole (CG2) vs. DSS + Metamizole (TG2) (°-°°°°); DSS + Water (TG1) vs. DSS + Metamizole (TG2) (*-****) were compared to each other using a Mann-Whitney-Test with the Holm-Šídák method. Within the groups, the change in score was compared Day 0 using the Wilcoxon signed-rank test to compare the score values with the baseline (hypothetical value of ‘0’ (CG1 (§), CG2 (°), TG1 (#) and TG2 (*)). Only a significance of p<0.0001 within the in-group analysis is shown on the graph. The n= 16 in each group until day 7, n=14 on day 8, n=12 on day 9 and n=11 on day 10 in TG1; n=12 on day 8, n=10 on day 9 and n=10 on day 10 in TG2. P was considered significant at ≤ 0.05 with * as p≤ 0.05; ** as p≤ 0.01; *** as p≤ 0.001; **** as p≤ 0.0001.

The clinical evaluation showed significantly higher scores in TG1 compared to CG1 between d4 and d9. Similarly, TG2 also showed significantly higher clinical scores as compared to CG2. In both groups, the highest value was observed on d7 (p = <0.0001). There were no significant differences observed in the clinical score between TG1 and TG2 (**Figure 8**).

The in-group analysis also showed a significant increase in the clinical scores of the mice between d4 and d9 in both the test groups when compared to their baseline scores on d0. In the TG2, a maximum score of 4.18 *(SD* = 3.31) and in TG1, the maximum score of 3.31 (*SD* = 1.99) was observed on d7. This was followed by a steady decline in the clinical score in both test groups until d10, indicating signs of recovery from the colitis. Both control groups (CG1 and CG2) showed no increase in the clinical scores and the values remained constant at “0” during the entire experiment.

**Figure 9:**
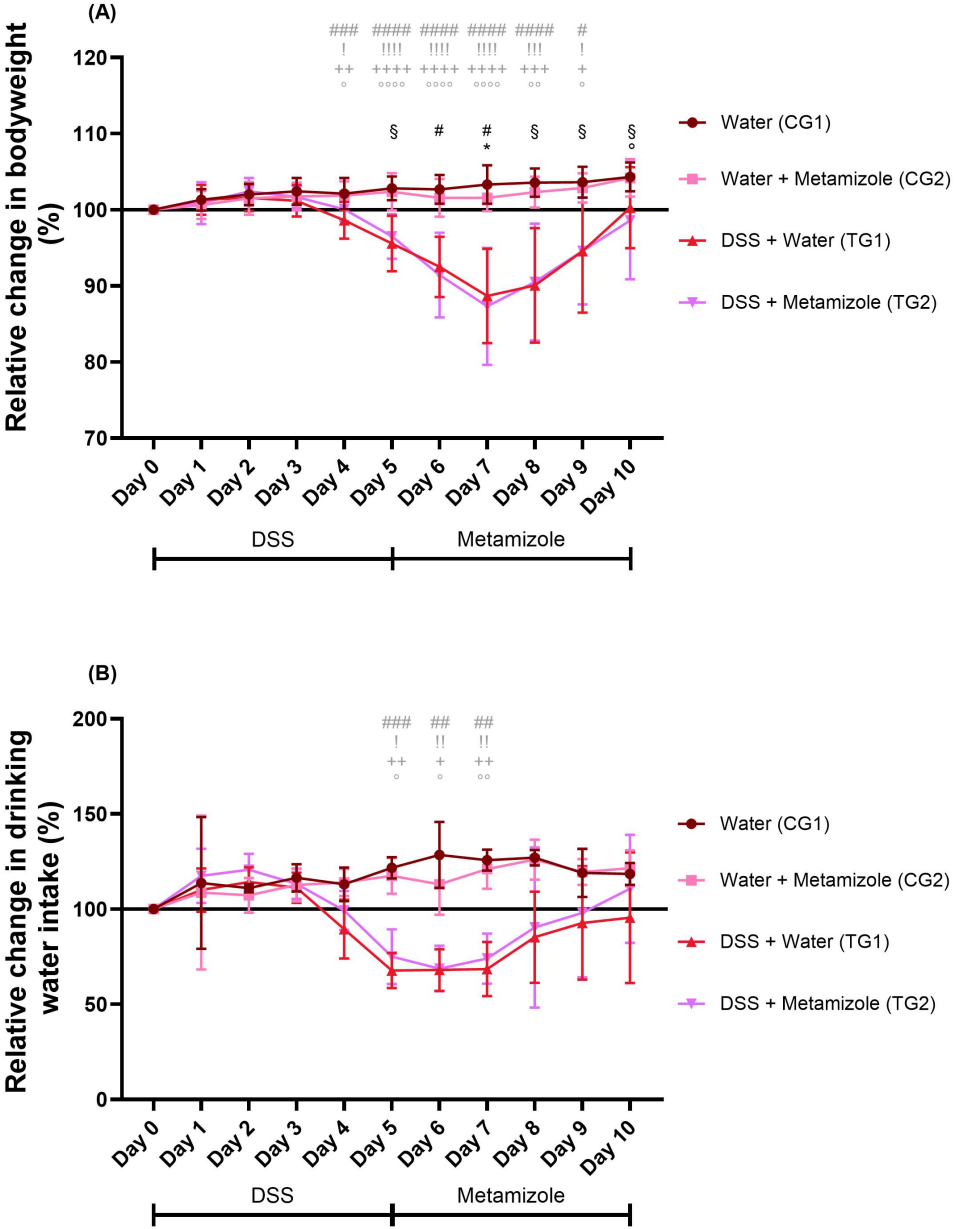
**(A)** Change in bodyweight during DSS and metamizole administration. Change in bodyweight compared to Day 0 (100%). The groups Water (CG1) vs. Water + Metamizole (CG2) (§-§§§); Water (CG1) vs. DSS + Water (TG1) (#-###); Water (CG1) vs. DSS + Metamizole (TG2) (!-)!!!; Water + Metamizole (CG2) vs. DSS + Water (TG1) (+-+++); Water + Metamizole (CG2) vs. DSS + Metamizole (TG2) (°-°°°); DSS + Water (TG1) vs. DSS + Metamizole (TG2) (*-***) were compared to each other using a Mixed-effects model (REML), Fixed effects (type III) followed by a Tukey’s multiple comparison test. Within the groups, the change in bodyweight was compared to the baseline using either a RM one way ANOVA with a Dunnett’s multiple comparison test (CG1 (§) and CG2 (°)) or a Mixed-effects model (REML), Fixed effects (type III) followed by a Dunnett’s multiple comparison test (TG1 (#) and TG2 (*)). Only a significance of p<0.0001 in the in-group analysis is shown on the graph. The n= 16 in each group until day 7, n=14 on day 8, n=12 on day 9 and n=11 on day 10 in TG1 (DSS+Water); n=12 on day 8, n=10 on day 9 and n=10 on day 10 in TG2 (DSS+Metamizole). P was considered significant at ≤ 0.05 with * as p≤ 0.05; ** as p≤ 0.01; *** as p≤ 0.001; **** as p≤ 0.0001. **(B)** Relative change in the intake of drinking water during DSS and metamizole administration. Change in the intake of drinking water was compared to the baseline (100%). The groups Water (CG1) vs. Water+Metamizole (CG2) (§-§§§); Water (CG1) vs. DSS + Water (TG1) (#-###); Water (CG1) vs. DSS + Metamizole (TG2) (!-)!!!; Water+Metamizole (CG2) vs. DSS + Water (TG1) (+-+++); Water+Metamizole (CG2) vs. DSS + Metamizole (TG2) (°-°°°); DSS + Water (TG1) vs. DSS + Metamizole (TG2) (*-***) were compared to each other using a Mixed-effects model (REML), Fixed effects (type III) followed by a Tukey’s multiple comparison test (grey symbols). Within the groups, the intake was compared to the baseline using a RM one-way Anova with a Dunnett’s multiple comparison test (CG1 (§), CG2 (°) and TG1 (#)) and a mixed-effects model (REML), Fixed effects (type III) followed by a Dunnett’s multiple comparison test (TG2 (*)). None of the groups showed a significance of p in the in-group analysis. The n=4 in all groups till day 7, after which n= 3 in TG2. P was considered significant at ≤ 0.05 with * as p≤ 0.05; ** as p≤ 0.01; *** as p≤ 0.001; **** as p≤ 0.0001.

One of the main clinical symptoms of acute DSS colitis is the loss of bodyweight which predominantly determines the clinical score results. Hence, the daily evaluation of the change in bodyweight during the DSS and subsequent metamizole administration is separately shown in **Figure 9A**.

#### 3.2.4 Drinking water uptake

The daily intake of drinking water (**Figure 9B**) of the group-housed mice was monitored by weighing the bottles and comparing the values to the baseline consumption (d0). On comparing the water control (CG1) with the group that received DSS and water (TG1), significant (p = 0.0064 - p = 0.0007) differences between the groups were observed due to a decline in drinking water intake in the DSS + water group (TG1) from days 5 to 7. In the analysis, significant differences (p = 0.0147 - p = 0.0064) were also seen in the DSS + metamizole (TG2) group when compared with the water + metamizole control (CG2) between days 5 to 6. The in-group analysis revealed a maximum reduction in the intake of drinking water in the DSS group (TG1) on day 6 with a 32% (*SD* = 10.98) decline from the baseline. In the DSS + metamizole group (TG2), a maximum reduction of 31.21% (*SD* = 11.95) was also observed on day 6. There were no significant differences observed in the intake of drinking water between both test groups (TG1 vs TG2) and both control groups (CG1 vs CG2). Thus, leading to the conclusion that the oral administration of metamizole via drinking water did not influence drinking water intake as seen in both control and test groups.

#### 3.2.5 Analysis of colon histology

An important characteristic of DSS-induced colitis is the histological damage caused to the colonic mucosa. Hence, to analyze the extent of colonic inflammation, a complete histological assessment of the colonic tissue was conducted by a blinded scorer.

**Figure 10:**
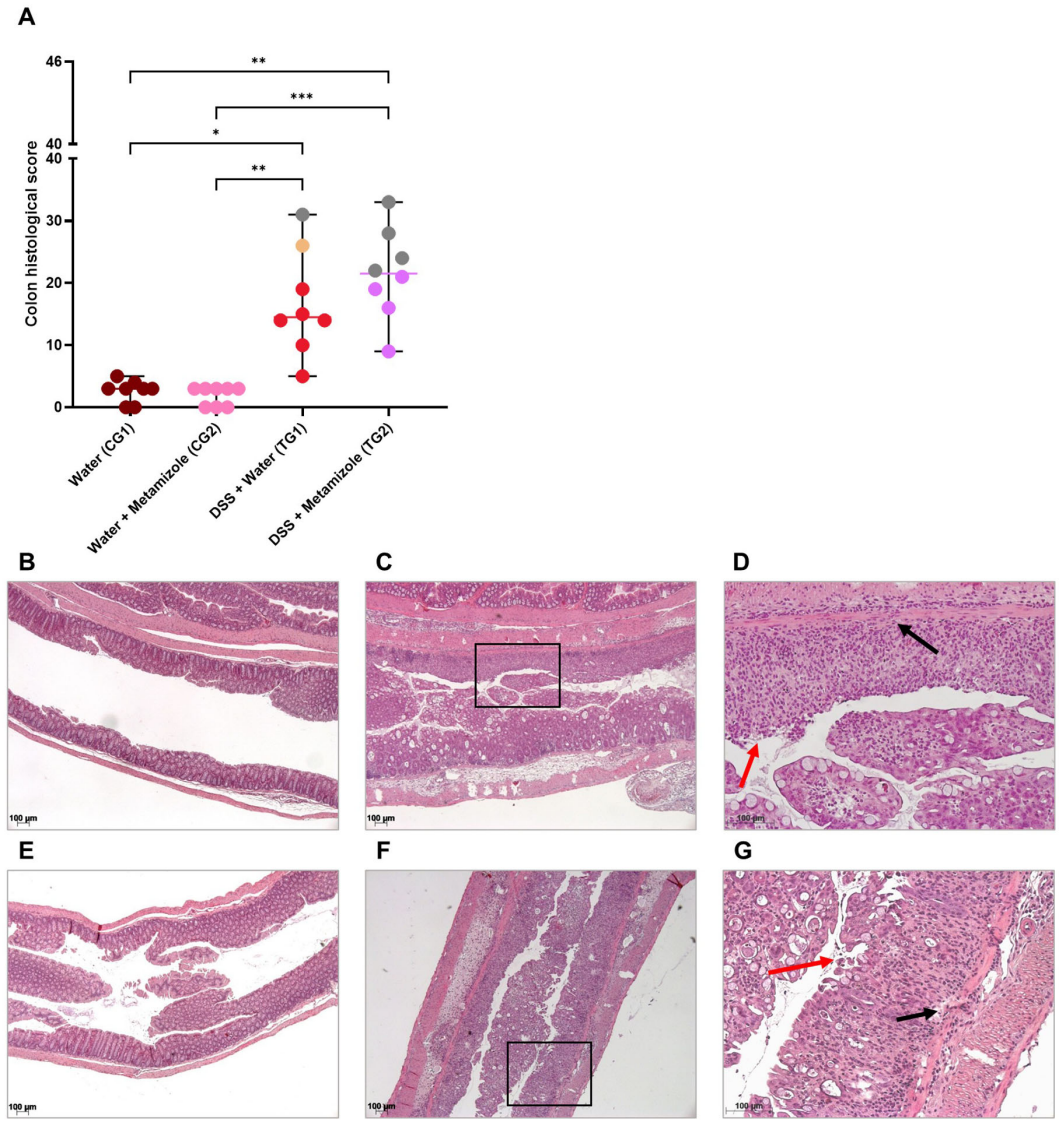
Colon histology of the four experiment groups. Samples were collected 10 days after starting the experiment (five days of DSS followed by five days of metamizole). The Kruskal-Wallis test followed by a Dunn’s multiple comparison was used to compare the groups with each other. The scores of the animals that were euthanized due to reaching the humane endpoint before the end of the experiment (TG1 and TG2 Days 9 and 7) are displayed with different colors on the graph. P was considered significant at ≤ 0.05 with * as p≤ 0.05; ** as p≤ 0.01; *** as p≤ 0.001; **** as p≤ 0.0001. The values on the graph are plotted as median with an IQR **(A)**. Representative HE stained histological slides of the four groups: Water (**(B)**: 5x); DSS + water [**(C)**: 5x; **(D)**: 20x]; water + metamizole [**(E)**: 5x]; DSS + metamizole [**(F)**: 5x; **(G)**: 20x]. Signs of acute colitis are evident through transmural neutrophil infiltration (black arrows) along with the loss of intestinal architecture and epithelial damage (red arrows) **(D, G)**.

The statistical analysis showed significantly higher scores in TG1 as compared to CG1 (p = 0.0263). TG2 also had significantly higher scores as compared to their water control (CG2) (p = 0.0003). On comparing both DSS groups (TG1 and TG2), the analysis did not detect any significant differences between the same, although TG2 had a higher median value of 21.5 (IQR 16.75-27) compared to TG1 with 14.5 (IQR 11-24.25). The highest scores (22–33 out of a possible 46) were observed in the mice that were euthanized due to meeting the humane endpoint criteria (highlighted in **Figures 10A–G**).

### 3.3 Part C: molecular biological analysis

To analyze the potential anti-inflammatory effect of metamizole on the DSS-induced colitis, a gene expression analysis of inflammatory cytokines and epithelial barrier proteins was carried out. Samples were collected on days with the highest disease severity, respectively on d7 and d10.

#### 3.3.1 Inflammatory cytokines

**Figure 11:**
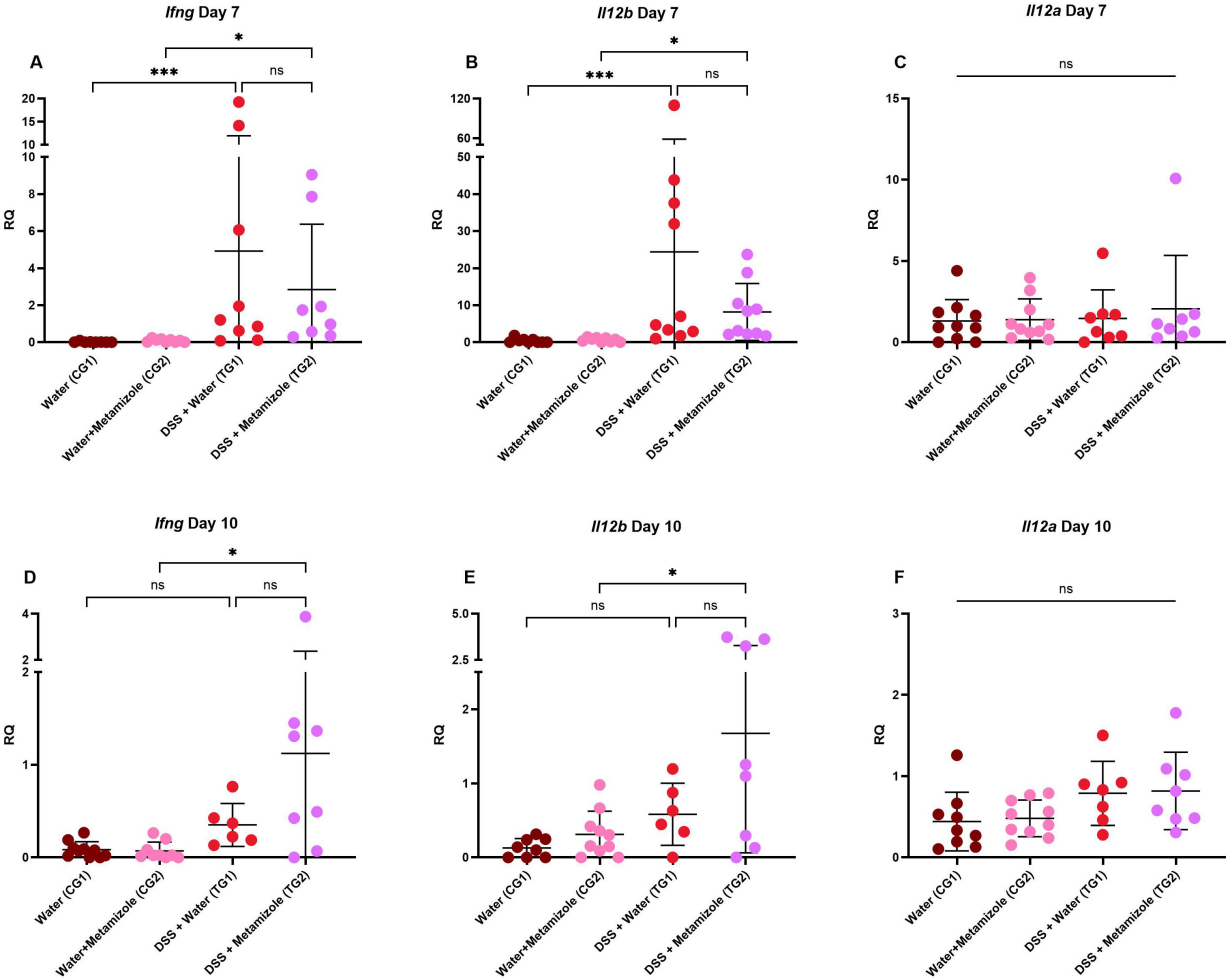
Gene expression analysis of pro-inflammatory cytokines on day 7 and day 10 following DSS and metamizole application. The gene expression of Ifng **(A, D)**, Il12b **(B, E)** and Il12a **(C, F)** were measured in the distal colon samples. The four experiment groups were compared to each other either using a two-way ANOVA followed by a Tukey’s multiple comparison for normally distributed data or a Kruskal-Wallis test followed by a Dunn’s multiple comparison test. Outliers were removed using the Rout method. The n=7–10 in each group after removing the outliers. Value points of mice that were euthanized before the end of the experiment are displayed in a different color. P was considered significant at ≤ 0.05 with * as p≤ 0.05; ** as p≤ 0.01; *** as p≤ 0.001; **** as p≤ 0.0001.

The gene expression analyses of the pro-inflammatory cytokines interferon gamma (*Ifng*) and interleukin 12b (*Il12b*) on day 7 revealed a significantly higher expression in both DSS groups as compared to their respective controls (**Figures 11A, B**), but no statistically significant differences were detected in the expression of interleukin 12a (*Il12a*) (**Figure 11C**). On the other hand, the samples collected on day 10 showed a significantly higher expression of *Ifng* and *Il12b* in TG2 compared to CG2 (**Figures 11D–F**). Interestingly, TG2 showed the trend of a higher expression of *Il12b* and *Ifng* than TG1 (**Figure 11E**).

**Figure 12:**
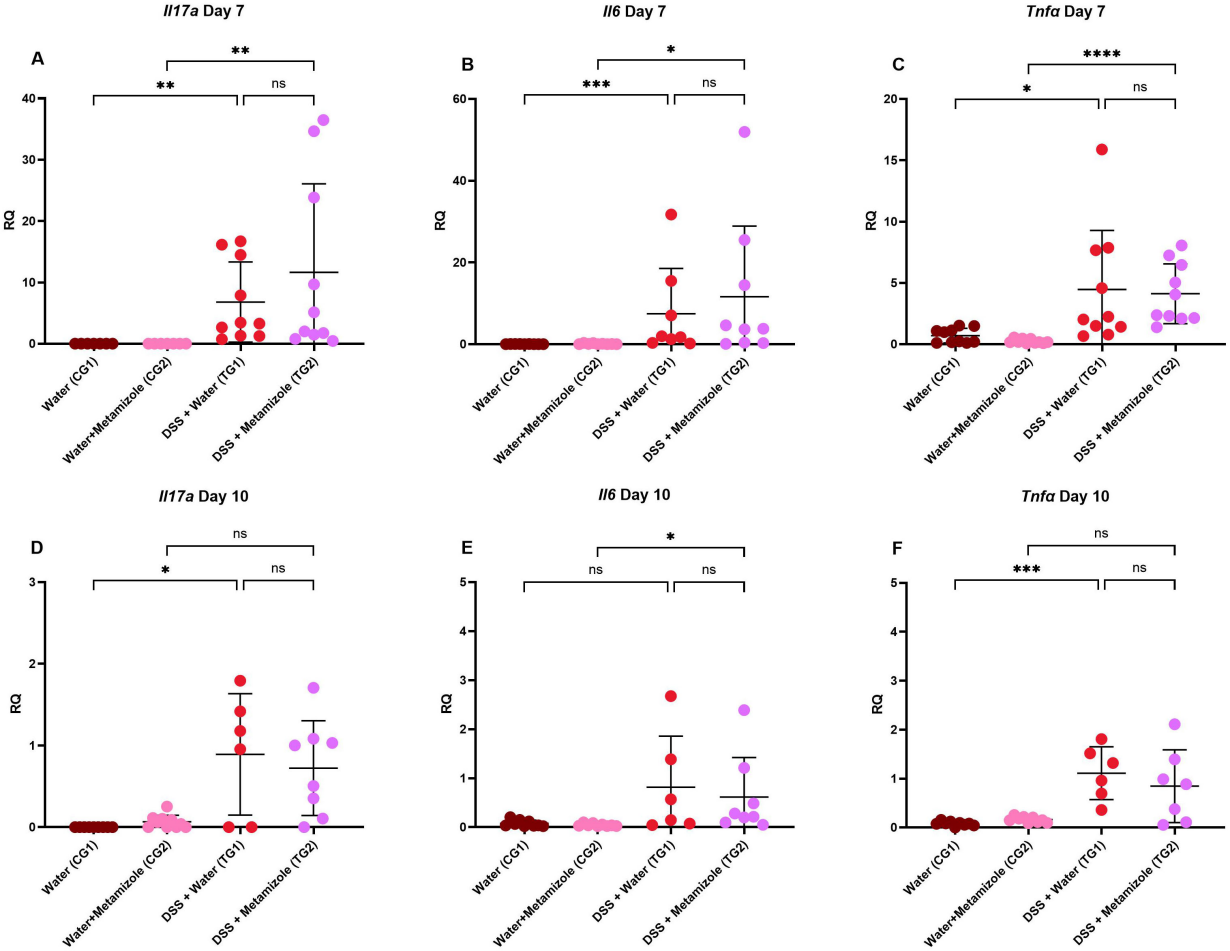
Gene expression analysis of pro-inflammatory cytokines on day 7 and day 10 following DSS and metamizole application. The gene expression of Il17a **(A, D)**, Il6 **(B, E)** and Tnfa **(C, F)** were measured in the distal colon samples. The four experiment groups were compared to each other either using a two-way ANOVA followed by a Tukey’s multiple comparison for normally distributed data or a Kruskal-Wallis test followed by a Dunn’s multiple comparison test. Outliers were removed using the Rout method. The n=7–10 in each group after removing the outliers. Value points of mice that were euthanized before the end of the experiment are displayed in a different color. P was considered significant at ≤ 0.05 with * as p≤ 0.05; ** as p≤ 0.01; *** as p≤ 0.001; **** as p≤ 0.0001.

Further analyses of interleukin 17a (*Il17a*), interleukin 6 (*Il6*) and tumor necrosis factor alpha (*Tnfa*) showed a significantly higher gene expression in TG1 and TG2 compared to CG1 and CG2 on day 7 (**Figures 12A–C**). On analyzing the samples collected on day 10, significantly higher expressions of *Tnfa* and *Il17a* were observed in TG1 compared to CG1 while there was a significantly higher expression of *Il6* in TG2 compared to CG2 (**Figures 12D–F**). However, the administration of metamizole did not lead to any significant difference in the gene expression analysis of the pro-inflammatory markers between TG1 and TG2.

#### 3.3.2 Epithelial barrier proteins

**Figure 13:**
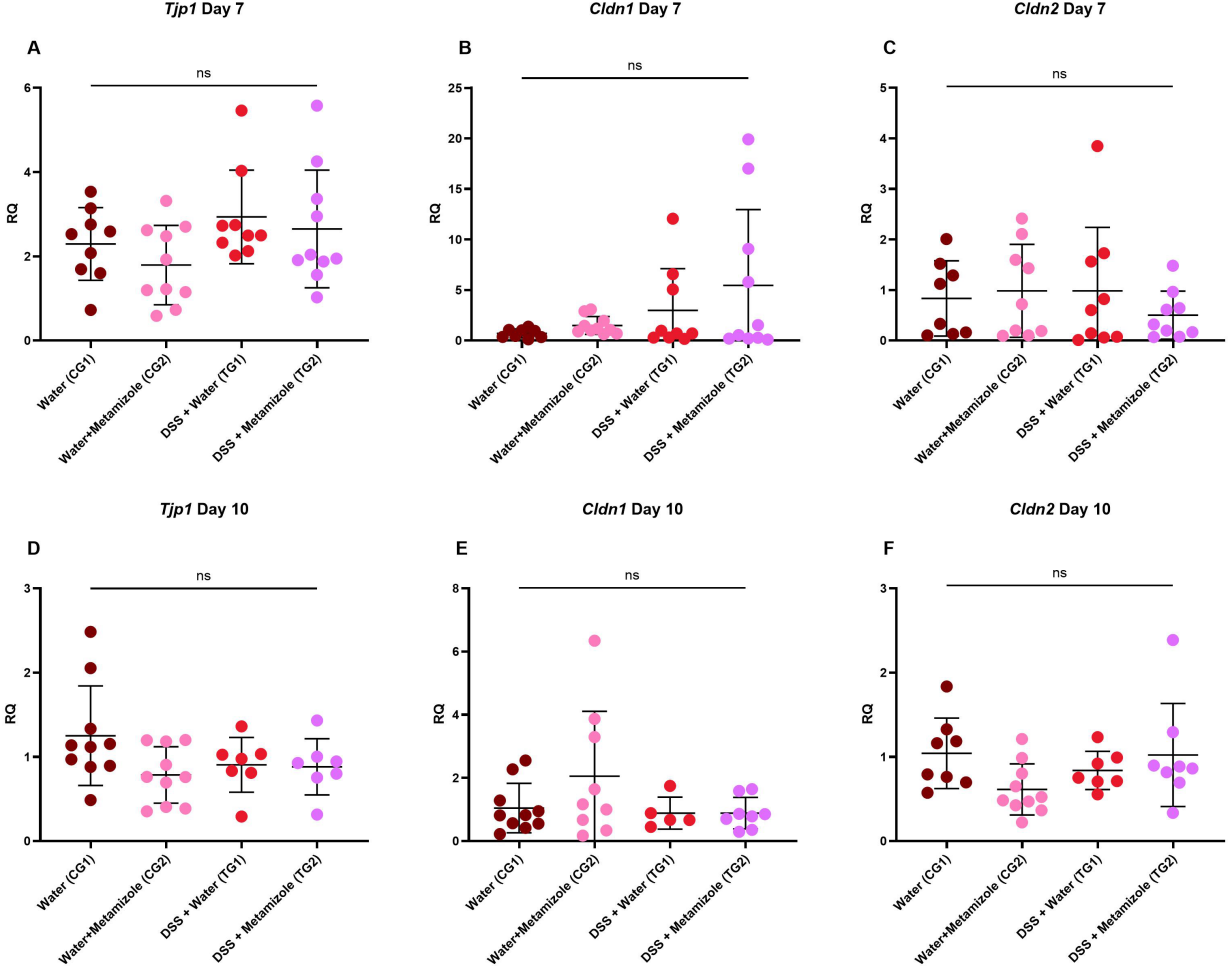
Gene expression analysis of tight junction proteins on day 7 and day 10 following DSS and metamizole application. The gene expression of Tjp1 **(A, D)**, Cldn1 **(B, E)** and Cldn2 **(C, F)** were measured in the distal colon samples. The four groups were compared to each other either using a two-way ANOVA followed by a Tukey’s multiple comparison for normally distributed data or a Kruskal-Wallis test followed by a Dunn’s multiple comparison test. Outliers were removed using the Rout method. The n=7–10 in each group after removing the outliers. Value points of mice that were euthanized before the end of the experiment are displayed in a different color. P was considered significant at ≤ 0.05 with * as p≤ 0.05; ** as p≤ 0.01; *** as p≤ 0.001; **** as p≤ 0.0001.

The integrity of the intestinal barrier was investigated through the expression of tight junction protein 1 (*Tjp1*) also known as Zonula occludens 1, a sealing claudin 1 (*Cldn1*) and the pore-forming claudin 2 (*Cldn2*) in the colon. The analysis however did not reveal any significant differences in the expression of these proteins between the control and the treatment groups (**Figures 13A–F**).

## 4 Discussion

According to legislation, pain, suffering, distress, and lasting harm imposed on laboratory animals need to be kept to an absolute minimum (2010/63/EU, 2010; Germany, 2006, 2013). This standard is met through refinement of experimental procedures and animal models. A central element of refinement is the use of analgesia to alleviate pain and improve overall well-being. The oral self-administration of the analgesic via drinking water was chosen to comply with general German guidelines (GV-SOLAS) and implement a stress-free method of administration. Against this background, we evaluated the analgesic effect of metamizole in a mouse model of DSS−induced acute colitis with the objective of refining the model.

In part A, we quantified the intake of metamizole via drinking water in DSS−induced colitis and measured plasma concentrations of the metabolites 4−MAA and 4−AA using LC–MS/MS. We focused on assessing behavior in part B by using VWR and MGS to determine whether metamizole reduces DSS−induced pain, thereby lowering experimental severity, and whether any improvement of well-being was reflected in VWR activity and MGS scores. Finally in part C, we examined intestinal inflammation by analyzing the expression of pro−inflammatory cytokines and tight junction proteins using qRT−PCR. The principal aim was to characterize the DSS colitis model following metamizole administration regarding refinement and to detect any anti−inflammatory effects that might compromise model integrity.

Although the results showed that metamizole was successfully delivered via drinking water, there was a substantial variability in metabolite plasma concentrations. Clinical and behavioral assessments showed no evidence of welfare improvement in DSS−treated mice receiving metamizole, suggesting that metamizole may be unsuitable for refining the DSS−induced acute colitis model under the conditions studied. Gene expression analysis of pro−inflammatory markers revealed only the trend of increased *Il12b* and *Ifng* expression in DSS−treated mice that received metamizole versus DSS alone (TG1 vs. TG2), with no significant differences in tight junction protein expression.

### 4.1 Metamizole: overview and use in animal studies

Metamizole (dipyrone) is a non−opioid analgesic marketed since 1922 and widely used in human and veterinary medicine for its antipyretic, spasmolytic, and analgesic properties (Hinz et al., 2007; Jasiecka et al., 2014; Levy et al., 1995). There have been various theories about the mechanism of action of metamizole, one of which is the inhibition of peripheral cyclooxygenase (COX) (Campos et al., 1999; Hinz et al., 2007). Chandrasekharan and colleagues showed a strong COX-3 inhibition of metamizole (Chandrasekharan et al., 2002). Metamizole is primarily indicated to treat spastic pain or pain in the gastrointestinal, urinary or biliary tract due to colic (Jasiecka et al., 2014; Levy et al., 1995). Owing to substantially fewer gastrointestinal side effects, it is often preferred over standard NSAIDs (Berenguer et al., 2002).

Metamizole is a prodrug and, on administration, rapidly hydrolyses to its active metabolite 4−methylaminoantipyrine (4−MAA). Hepatic cytochrome P450 enzymes further metabolize 4−MAA to its terminal metabolites, notably 4−aminoantipyrine (4−AA) (Levy et al., 1995; Lutz, 2019; Noda et al., 1976; Rogosch et al., 2012). After oral consumption in humans, the parent drug (metamizole/dipyrone) is undetectable in plasma and urine; following intravenous administration, it is detectable in serum for approximately 15 minutes (Vlahov et al., 1990). Hence, the analgesic effect of metamizole is presumed to correlate with the concentration of these metabolites (Rohdewald et al., 1983). In the present study, the LC-MS/MS analysis of the plasma samples revealed highly variable concentrations of the metabolites 4−AA and 4-MAA. This could be attributed to several factors that influence the pharmacokinetic distribution of the drug, including blood flow, its protein-binding ability, and its ability to penetrate the blood–brain barrier. The health status and age also play an important role in how a drug is metabolized (Currie, 2018). It has been shown that patients suffering from liver disease took longer to eliminate metamizole from their system (Hinz et al., 2007; Lupu et al., 2022). Therefore, the result of a disrupted hepatic lipid metabolism during DSS-induced colitis might also be a confounding factor in the present study (Kwon et al., 2021).

Another confounder could be the lower intake of drinking water due to DSS-treatment. Generally dehydrated mice are characterized by having a higher plasma osmolality, increased packed cell volume, and total plasma protein (Bae et al., 2006; Bekkevold et al., 2013). These factors could also potentially hamper with the pharmacokinetic effects of the metabolites.

Pharmacokinetic studies of metamizole metabolites have been conducted for other species like sheep (Giorgi et al., 2015), donkeys (Aupanun et al., 2016), dogs (Giorgi et al., 2018), cats (de Paula et al., 2023) and also rats (Carrillo-Calzadilla et al., 2017). However, to our knowledge there is no published data addressing the therapeutic dose or plasma concentration of 4-MAA and 4-AA in healthy mice.

Evidence for antinociceptive efficacy in animals, particularly mice, remains limited and inconclusive (Jasiecka et al., 2014; Lupu et al., 2022). Nevertheless, a review of animal research proposals reported metamizole use as perioperative analgesia in 8–9% of cases and as the third most common analgesic for postoperative pain in rodents (Herrmann, 2019).

There a several studies investigating the effects of metamizole treatment on pain. In a study by Stumpf and colleagues, metamizole was evaluated as an analgesic to relieve pain in a mouse model of cerulein-induced acute pancreatitis. Here, metamizole was administered orally 4 times at a dose of 150 mg/kg, leading to reduced central pain responses (Stumpf et al., 2016). In another study, intraperitoneal administration of metamizole (250 mg/kg) was also shown to effectively attenuate visceral pain in mice, as demonstrated by a significant reduction in writhing behavior induced by acetic acid (Taylor et al., 1998).

When comparing the frequency and route of oral administration in these studies with the oral self-administration of metamizole via drinking water as carried out in the present study, the observed analgesic effect could potentially be attributed to the more precise route of administration used by the authors. On the other hand, the process of restraining the mice for the oral or intraperitoneal administration is also associated with increased stress for the mice.

The studies by Stumpf and Taylor do not provide information on the pharmacokinetics of metamizole metabolites. Furthermore, differences in animal models, routes of administration, and dosages should be considered when comparing the outcomes of these studies with the results of the present study.

Overall, oral self-administration of analgesics is controversial due to potential unreliability in achieving the required dosage, especially in group-housed mice (Jirkof et al., 2018). Other factors that may influence intake include circadian rhythm (Stephan and Zucker, 1972), neophobia or aversion to the analgesic taste (Speth et al., 2001), and the animal’s health status and pain (Jirkof et al., 2018; Liles et al., 1998). Despite these concerns, voluntary analgesic intake via drinking water provides a stress-free administration method and has been successful in multiple studies (Glasenapp et al., 2024; Jessen et al., 2007; Liles et al., 1998; Sauer et al., 2016).

In studies using laboratory animals, metamizole is primarily administered orally at 200 mg/kg/day as recommended by the GV-SOLAS (Margarete Arras et al., 2020). However, some studies reported reduced water intake during metamizole self-administration in mice, leading to weight loss (Durst et al., 2021; Spalinger et al., 2023). Spalinger et al. observed near-complete water refusal with metamizole plus DSS in the last four days of a 7-day course; adding sugar led to a modest increase in intake, which overall remained low (Spalinger et al., 2023). Durst et al. found lower and more variable metamizole intake in male C57BL/6J mice compared with buprenorphine-containing water or paracetamol with tramadol. In the present study, no significant differences were found between metamizole and water controls (**Figure 9B**). Metamizole intake also varies by mouse strain, requiring strain-specific habituation before oral administration (Schreiber et al., 2025).

Pain also influences mice water intake. Surgical pain and DSS-induced symptoms reduce intake, potentially preventing analgesics from reaching the target dose (Sauer et al., 2016). In Part A of the present study, water intake declined with DSS symptoms and during metamizole administration, then rose toward baseline by day 7. Here it needs to be considered that day 7 involved two blood draws and cage changes, causing additional water loss (**Supplementary Figure 7**). In Part B, a similar decline occurred, with the largest drop between days 6 and 7 (**Figure 9B**) coinciding with the highest clinical scores and decline in bodyweight observed on day 7 (**Figures 8**, **9A**). On analyzing the voluntary intake of metamizole via drinking water using the change in individual bodyweight and drinking water intake of the group between CG2 and TG2, the mice that received DSS (TG2) showed a significantly lower intake of metamizole with a mean dose of 148.6 mg/kg/day (*SD* = 18.6) on day 6 compared to the controls (CG2) with a mean dose of 197.1 mg/kg/day (*SD* = 17.8). This was followed by an increase with a mean metamizole intake of 278.8 mg/kg/day (*SD* = 45.2) on day 10 in TG2. (**Supplementary Figure 8**).

Sex- and strain-specific differences in pain perception and analgesic responsiveness are increasingly recognized in laboratory mice and should be acknowledged when interpreting findings generated exclusively in female C57BL/6J mice. Numerous studies demonstrate that both genetic background and sex strongly influence nociceptive sensitivity as well as responses to analgesic agents, leading to measurable behavioral differences across strains and between males and females. In addition, evidence shows that sex interacts with genotype and environmental factors, with some strains—including C57BL/6—displaying distinct male–female differences in analgesic outcomes or pain-related mechanisms. These findings highlight the importance of considering sex as a biological variable, as reliance on a single sex may limit the generalizability of results and obscure relevant biological variation in pain pathways.

However, the gap in literature and the lack of information regarding the therapeutic level for metamizole’s analgesic efficacy in mice complicate the interpretation of the obtained results. It cannot be determined whether the chosen route of oral self-administration failed to produce an analgesic effect because the necessary therapeutic dose was not reached, or whether metamizole is ineffective as an analgesic in the mouse model of DSS-induced acute colitis. While the study provided valuable insights into the voluntary oral intake of metamizole via drinking water in group-housed mice, the inability to determine the individual intake and dose received by each animal represents a major limitation with respect to assessing analgesic efficacy in the experimental model. In this regard, the study may have benefited from the use of a more precise route of analgesic administration. Sex- and strain-specific differences in pain perception and analgesic responsiveness are increasingly recognized in laboratory mice and should also be acknowledged when interpreting findings generated exclusively in female C57BL/6J mice (Smith, 2019). Our analysis also did not reveal a correlation between plasma concentrations of the metabolites and the clinical score, including bodyweight change, general appearance, and activity levels. Therefore, further pharmacokinetic investigations and assessments of the antinociceptive efficacy of metamizole metabolites in mice are warranted in future analgesia studies.

### 4.2 Behavioral assessment of pain: VWR and MGS

Some studies show evidence of abdominal pain in mice as a result of visceral hypersensitivity due to DSS- induced colitis in this model (Eijkelkamp et al., 2007; Vezza et al., 2023; López-Estévez et al., 2021). In this context, burrowing has been described as a pain-detection parameter in the DSS-induced colitis mouse model, with altered burrowing activity accompanying symptom onset (Jirkof et al., 2013; Struve, 2020). As burrowing requires single housing of mice, which per se is a compromising factor in the well-being of the animals, this method was not chosen in this study. Earlier investigations by our research group showed that VWR is the most sensitive and robust method for detecting disturbed welfare in the DSS colitis model (Häger et al., 2018; Weegh et al., 2020). Therefore, VWR together with the MGS, considered the gold standard for measuring pain, were selected parameters in the present study.

### 4.3 VWR

Voluntary wheel running is a highly sensitive behavioral parameter for measuring activity in mice and is biologically distinct from general activity (Sherwin, 1998). For pain assessment, voluntary wheel running has been used and validated across numerous models of surgery, inflammation, and disease. In a study by Tubbs and colleagues, continuous wheel running activity was employed to evaluate the effects of various analgesics in mice after partial hepatectomy (Tubbs et al., 2011), while Helwig and colleagues used wheel running to examine post-surgical recovery in mice after intraperitoneal implantation of radio telemetry devices of varying sizes (Helwig et al., 2012). VWR has been used to quantify pain−related behavior in models of peripheral inflammation, including intraplantar Complete Freund’s Adjuvant (CFA) injections (Cobos et al., 2012; Contreras et al., 2021), and to assess severity in disease models such as pancreatic cancer (Weegh et al., 2021) and DSS−induced colitis (Häger et al., 2018; Weegh et al., 2020). In the present study, VWR activity declined significantly in DSS−treated mice until day 7 and then increased toward the end of the experiment, consistent with findings from those previous studies. However, unlike the clinical parameters, total distance travelled and daily maximum speed failed to return to baseline by the end of the study, remaining approximately 30–55% below baseline (**Figures 5**, **6**). This suggests residual stress or impaired well−being due to inflammation that was not captured by clinical scoring. It has already been observed that VWR can reveal disturbed well−being even when clinical evaluation does not, suggesting that VWR is a more sensitive indicator than clinical scoring (Häger et al., 2018; Weegh et al., 2020; Zentrich, 2022).

Nevertheless, in this study metamizole did not alter VWR activity: no significant differences were observed between DSS−treated mice receiving metamizole and those receiving water (DSS + water vs. DSS + metamizole) (**Figures 5**, **6**).

### 4.4 MGS

We also evaluated the MGS developed by Langford et al (Langford et al., 2010), which has been applied across acute, chemical, procedural, inflammatory, post−surgical, neuropathic, and disease−related pain models. as summarized by Mogil et al (Mogil et al., 2020). Matsumiya and colleagues used MGS to compare four analgesics in a laparotomy model: buprenorphine showed the greatest efficacy, even at doses below typical recommendations; carprofen and ketoprofen were effective only at higher−than−recommended doses and acetaminophen (paracetamol) showed no effect on postoperative pain (Matsumiya et al., 2012).

In the present study, median baseline MGS scores ranged from 0.58 to 0.81 across the four groups, consistent with Miller and Leach’s observation that baseline MGS is not zero (Miller and Leach, 2015). A retrospective analysis also comes with its own set of challenges including organizing huge amounts of data generated while collecting footage. To address this issue, fully automated (Tuttle et al., 2018) and semi−automated (Sotocina et al., 2011) tools have been developed to simplify scoring (Mogil et al., 2020). For example, the cloud−based PainFace software detects changes in facial action units (AUs) in black−coated mice and assigns scores (McCoy et al., 2024).

Here, two blinded scorers used a previously published semi−automated software (Ernst et al., 2020). Footage was acquired by recording mice in observation cubicles for a defined period. Overall, no significant differences were detected between experimental groups (**Figure 7**). DSS−treated mice did not exhibit significantly elevated MGS scores, even on days with maximal clinical scores. Even in animals reaching humane endpoints (>20% weight loss), high clinical scores due to hunched posture and reduced activity were not mirrored in MGS scores. Recent findings indicate that the MGS shows limited sensitivity for detecting visceral pain, particularly in models of intestinal inflammation. While the MGS reliably identified post−operative pain, with elevated scores occurring shortly after abdominal surgery, it did not detect increased pain scores during DSS−induced colitis or *Citrobacter rodentium* infection, despite clear signs of reduced wellbeing such as weight loss and elevated clinical scores (Peppermüller et al., 2023); while live scoring is less sensitive than retrospective scoring (Miller and Leach, 2015). In a recent study testing amantadine for analgesia in a DSS-colitis mouse model, the authors stated, that not all DSS-treated animals showed signs of pain and the reported MGS was also very low (Barleben et al., 2025). Similar outcomes were observed in a study assessing the affective state in a mouse model of colitis-associated colorectal cancer. In that study, the analysis did not reveal significant differences in the MGS, although the authors noted non-facial indicators of pain—such as abdominal twitching, hunching, writhing, and belly pressing—during the experimental period (Chartier et al., 2020). This pattern suggests that visceral or gastrointestinal pain may not elicit robust facial action unit changes, or that such changes are too subtle to be captured by the MGS under these conditions.

An additional discussion point concerns the strain and coat color of the mice in relation to a retrospective analysis. The MGS originally developed by Langford and colleagues was described in mice with white fur, and assigning scores to black-coated mice proved difficult, particularly for inexperienced scorers (Hohlbaum et al., 2020). Moreover, Hohlbaum and colleagues reported that the nose and cheek bulge were the two FAUs with the lowest reliability in black-furred mice due to reduced contrast and difficulty in recognizing these facial features compared with other FAUs (Hohlbaum et al., 2020). Since McCoy and colleagues reported similar observations, we excluded cheek bulge from our analysis (McCoy et al., 2024). Taken together, these observations raise the question of whether the MGS is the most appropriate test for assessing visceral pain in the DSS-induced acute colitis mouse model. The diffuse nature of visceral pain may also hinder MGS analysis in this model. As in human IBD patients, alleviating visceral pain presents challenges related to identifying the exact source/location of the pain (Sikandar and Dickenson, 2012). Chartier and colleagues discuss that, by nature, mice are prey animals with an instinct to conceal weakness, which could contribute to discrepancies when recording MGS videos (Chartier et al., 2020). The question remains, therefore, whether DSS-treated animals actually experience higher levels of pain, or whether the MGS method is at all suitable for pain assessment in this model.

### 4.5 Gene expression

Changes in the inflammatory features of the model, due to metamizole treatment, may inadvertently lead to false outcomes and thus hamper with the translatability to human studies. To address this issue, we conducted a gene expression analysis of various pro-inflammatory cytokines and tight junction proteins to gain an insight on the inflammation and integrity of the epithelial barrier after a five-day DSS administration followed by a treatment with metamizole. For our analysis, samples of the distal colon were taken on d7 and d10 after beginning the DSS administration.

Many cytokines play a role in the inflammatory process of IBD and have been detected in higher levels in patients suffering from the disease (Niessner and Volk, 1995; Parronchi et al., 1997). In the DSS-induced mouse model of colitis, Dieleman and colleagues intimated the involvement of both Th1 and Th2 cells in the development of chronic colitis (Dieleman et al., 2001). However, in later studies, the role of Th17 cells was also acknowledged in the pathogenesis of IBD (Miossec and Kolls, 2012).

While our analysis revealed no differences in tight junction protein expression, significantly elevated expressions of the pro-inflammatory cytokines *Ifng*, *Tnfa*, *Il12b*, *Il17a* and *Il6* were detected in the colonic tissue samples from DSS-treated mice collected on day 7 compared to water controls. These results align with previous studies showing enhanced expression of pro-inflammatory cytokines after DSS administration (Yan et al., 2009). However, no significant differences were observed in the gene expression of DSS-mice treated with metamizole (TG1 vs TG2). On day 10, *Ifng*, *Il12b*, and *Il6* were higher in metamizole-treated groups after DSS (TG2) than controls that received metamizole only (CG2). *Il17a* and *Tnfa* were higher in DSS-only mice (TG1) versus water controls (CG1). Notably, on day 10, the expression of *Il12b* was higher in DSS and metamizole treated mice (TG2) as compared to mice treated with DSS and water only (TG1), indicating a potential effect of metamizole.

A study by Eftychi and colleagues has described *Il12* to play an important role in colonic inflammation during the early stages of IBD and this role being taken over by *Il23* during the chronic phase of the disease (Eftychi et al., 2019). In our study, the high expression of Il12b on day 10 in TG2 could potentially indicate an effect of metamizole on the regeneration process during the period of recovery. This suggestion is further supported by a study of Spalinger and colleagues, where they observed higher expressions of *Il6* and *Il1b* in mice of a chronic DSS model treated with metamizole (Spalinger et al., 2023).

### 4.6 Outlook

Bridging the gap in the literature by addressing the pharmacological characteristics and therapeutic levels of metamizole in healthy mice would be beneficial for future studies. The current study may have also benefitted with a direct comparison of metamizole to other analgesics or by analyzing the effect of administering metamizole in combination with other analgesics. Furthermore, analyzing the intake of metamizole by monitoring the metabolite concentrations at various time-points may give us more clarity on the intake during the different stages of the disease and whether the model could benefit from an earlier administration of metamizole or an additional intervention at a different time point.

From a refinement standpoint, investigating the use of other non-invasive and more precise methods of analgesic administration could be considered for the DSS-colitis model as well as other IBD models. Finally, exploring sex- and strain-specific differences would also be of great value to future studies.

## 5 Conclusion

Regarding the behavioral analysis and given the lack of published data on the therapeutic level of metamizole, it cannot be definitively assessed whether orally administered metamizole via drinking water had an analgesic effect on the mice, as voluntary water intake in group-housed mice might lead to variable drug exposure. Another question is whether DSS-administered mice actually experience pain. The results of the VWR analysis showed that treatment with DSS led to a reduction in activity. By contrast, the MGS was not elevated at any time. At this stage, it can only be speculated that the MGS does not detect visceral pain, whereas the VWR does.

However, while prior studies and results of the present study suggest the development of pain after DSS administration, we cannot make a definitive statement that the mice in this model are in continuous pain due to intestinal inflammation. The absence of a positive control analgesic also limits the interpretation of the results, as it is unclear whether pain-related behavior can be reliably detected in this model or whether said results point to the lack of analgesic efficacy of metamizole.

In conclusion, further studies are required to explore new methods to refine animal models and thus abide by the 3R-principle.

## Data Availability

The raw data supporting the conclusions of this article will be made available by the authors, without undue reservation.
