# Supplementary material for: Evaluating the analgesic effect of metamizole in the mouse model for DSS-induced acute colitis
Source: Front Physiol. 2026 Aug 5;17:1810607. doi: 10.3389/fphys.2026.1810607 (PMC13485539; doi:10.3389/fphys.2026.1810607)
Supplement: Supplementary file 1 [file DataSheet1.pdf]

Supplementary table 1: Clinical Score Sheet

Parameter	Criteria	Recommended course of action	Score
Activity	active, alert, quick movements, social interaction		0
	reduced activity, less alert, occasional interruption in movement; social interaction		1
	reduced activity, mouse is still, social interaction	Animal needs to be examined and weighed twice daily<br>Improvement: Increase in activity	2
	inactive, self-isolation	No-go criterion, euthanize affected animal	3
Bodyweight loss	<5%		0
	5-9,9%		1
	10-14,9%		2
	15-19,9%	Animal needs to be examined and weighed twice daily<br>Improvement: No further reduction or an increase in bodyweight	3
	≥20%	No-go criterion, euthanize affected animal	4
General condition	Fur: smooth, glossy; Eyes: clear, glossy		0
	Fur: smooth, glossy; Eyes: clear, glossy; slightly hunched (protective/relieving) body posture		1
	Change in fur (ruffled, reduced self-grooming); hunched body posture; Eyes: squinted	Animal needs to be examined and weighed twice daily<br>Improvement: Improvement in fur and/or body posture	2
	Soiled fur; pathological discharge from body orifices; increased muscle tone	No-go criterion, euthanize affected animal	3
Stool consistency	Solid		0
	Solid to soft		1
	Solid to soft with traces of blood		2
	Very soft with traces of blood	Animal needs to be examined and weighed twice daily<br>Improvement: reduction in the amount or no more blood observed	3
	Severe, bloody diarrhoea	No-go criterion, euthanize affected animal	4
Wound healing	No redness or swelling		0
	Redness or swelling	Treatment with Iodine-ointment	1
**Total Score**			**15**

Supplementary table 2: Training protocol for the acclimatization of the mice to the MGS observation boxes

Prior to training: place observation box in home cage for 10-12 minutes of acclimatization	
Date	Handling instructions
Training Session 1	Mouse to be placed in the box for a maximum of 10 seconds
Training Session 2	Mouse to be placed in the box for one to three minutes
Training Session 3	Mouse to be placed in the box for a maximum of 10 minutes inside the illuminated photo tent

Supplementary table 3: Histological scoring parameters based on Häger et. Al., 2015.

Inflammatory Cells	Severity	1 = mild<br>2 = moderate<br>3 = marked<br>4 = severe
	Maximum extent	1 = Mucosa, L. propria<br>2 = 1 + Submucosa<br>3 = 2 + transmural, L. muscularis<br>4 = 3 + breakthrough, Peritonitis
Intestinal Architecture	Epithelial	1 = focal erosions<br>2 = marked erosions<br>3 = several erosions<br>4 = extended ulcerations
	Mucosal	1 = focal architecture loss, blunted crypts<br>2 = moderate architecture loss, mucine retention<br>3 = marked architecture loss, crypt necrosis<br>4 = no architecture
Extent of Edema		1 = edema in epithelial cells<br>2 = edema in submucosa<br>3 = edema in muscular layers
Area involved		1 25%<br>2 50%<br>3 75%<br>4 100%

Supplementary table 4: Primer list (TaqMan® Gene Expression Assay)

Gene Name	Gene Symbol	Assay ID
Eukaryotic translation elongation factor 2	*Eef*2	Mm01171434_g1
Interferon gamma	*Ifng*	Mm01168134_m1
Interleukin 6	*Il6*	Mm00446190_m1
Interleukin 12a	*Il12a*	Mm00434169_m1
Interleukin 12b	*Il12b*	Mm99999067_m1
Interleukin 17a	*Il17a*	Mm00439619_m1
Tumor necrosis factor alpha	*Tnfa*	Mm00443258_m1
Zonula 1 (Tight junction protein 1)	*Tjp1*	Mm01320638_m1

Supplementary table 5: qPCR Reaction Mix for TaqMan® Gene Expression Analysis (Volume for 1 reaction)

Component	Volume
TaqMan® Fast advanced Master Mix	10 µl
TaqMan™ Gene Expression Assay (20X)	1 µl
Sterile water	7 µl
cDNA/Water	2 µl
**Total volume**	**20 µl**

Supplementary table 6: Thermal protocol for TaqMan® Gene Expression Analysis

Step	Temperature	Time	Cycles
Enzyme activation	95˚C	20 sec	1
Denature	95˚C	1 sec	40
Anneal/Extend	60˚C	20 sec	

Supplementary table 7: Primer list (Sybr®Green Gene Expression Analysis; QuantiTect®)

Gene Name	Gene Symbol	Assay ID
Claudin 1	*Cldn*1	Mm_Cldn1_1_SG
Claudin 2	*Cldn*2	Mm_Cldn2_2_SG

Supplementary table 8: Housekeeping primer (Sybr®Green Gene Expression Analysis; Eurofins Genomics™)

Gene Name	Gene Symbol	Primer 5’→3’
Eukaryotic translation elongation factor 2	*Eef*2 for	TGTCAGTCATCGCCCATGTG
Eukaryotic translation elongation factor 2	*Eef*2 rev	CATCCTTGCGAGTGTCAGTGA

Supplementary table 9: qPCR Reaction Mix for Sybr®Green Gene Expression Analysis (Volume for 1 reaction)

Component	Volume
Sybr®Green Fast Master Mix	10 µl
Sybr®Green primer	2.5 µl
Sterile water	5.5 µl
cDNA/Water	2 µl
**Total volume**	**20 µl**

Supplementary table 10: Thermal protocol for Sybr®Green Gene Expression Analysis

Step	Temperature	Time	Cycles
Hold stage	95˚C	20 sec	1
PCR stage	95˚C	1 sec	40
	60˚C	20 sec	
Melt stage	95˚C	15 sec	1
	60˚C	60 sec	
	95˚C	15 sec	
