# Supplementary material for: Evaluating the analgesic effect of metamizole in the mouse model for DSS-induced acute colitis
Source: Front Physiol. 2026 Aug 5;17:1810607. doi: 10.3389/fphys.2026.1810607 (PMC13485539; doi:10.3389/fphys.2026.1810607)

Supplementary Figure 1. Change in bodyweight during DSS and metamizole application. For the analysis, we used a linear mixed effects model; Fixed effect type III: p value <0.0001, F (1.954, 29.02) = 28.30 with a Dunnett's multiple comparisons test ($p =< 0.05$). (n=16: days 1-6; n=15: Day 7). P was considered significant at ≤ 0.05 with * as p≤ 0.05; ** as p≤ 0.01; *** as p≤ 0.001; **** as p≤ 0.0001.

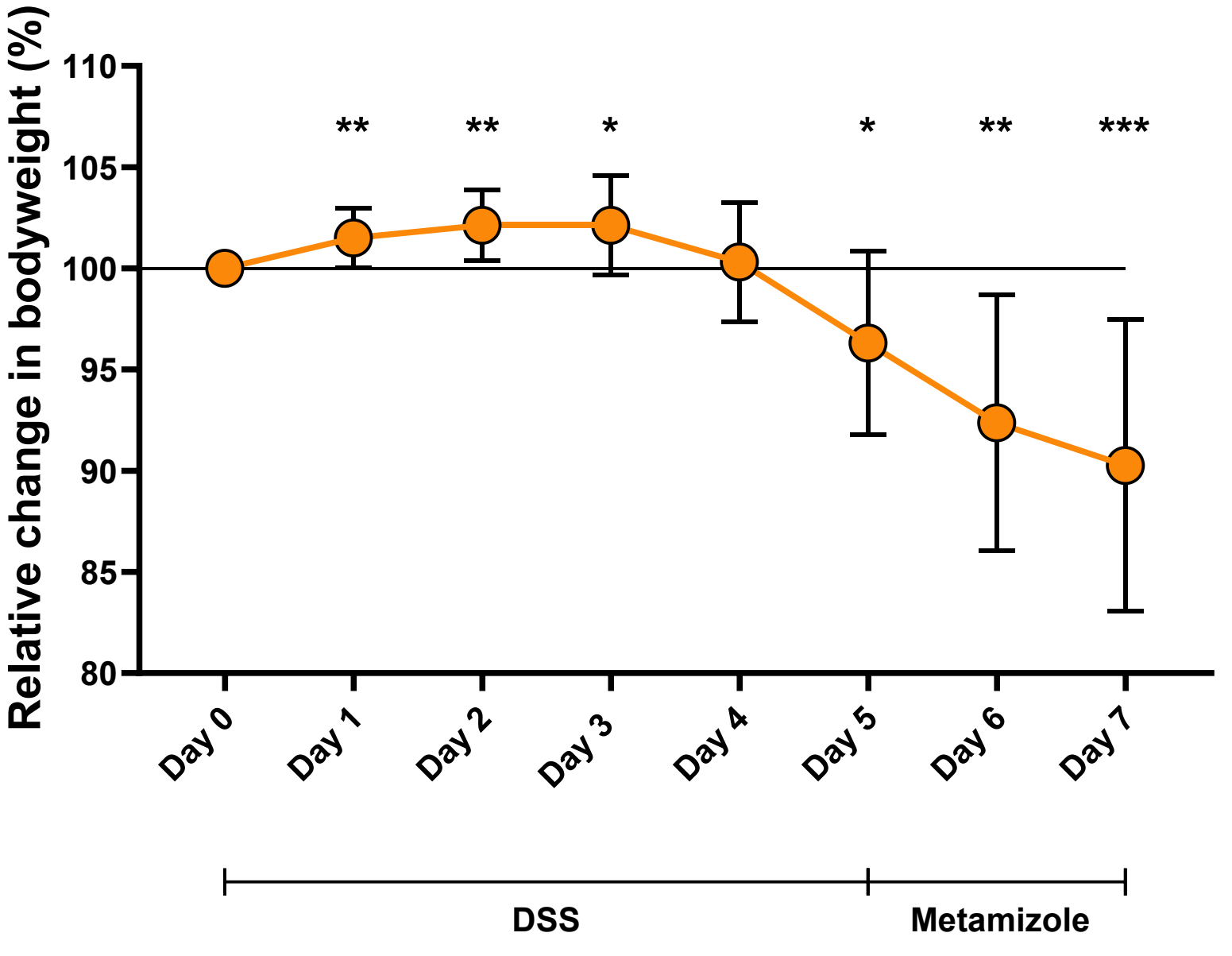

Supplementary Figure 2. Total clinical scores and colon histology. Clinical scores were compared to Day 0 values using Wilcoxon Signed Rank Test ($p =< 0.05$) (n=16: days 1-6; n=15: Day 7) (A). The histological score ranges from 0-46 and the results revealed a mean score of 19.07±6.307 SD (n=15) (B). P was considered significant at ≤ 0.05 with * as p≤ 0.05; ** as p≤ 0.01; *** as p≤ 0.001; **** as p≤ 0.0001.

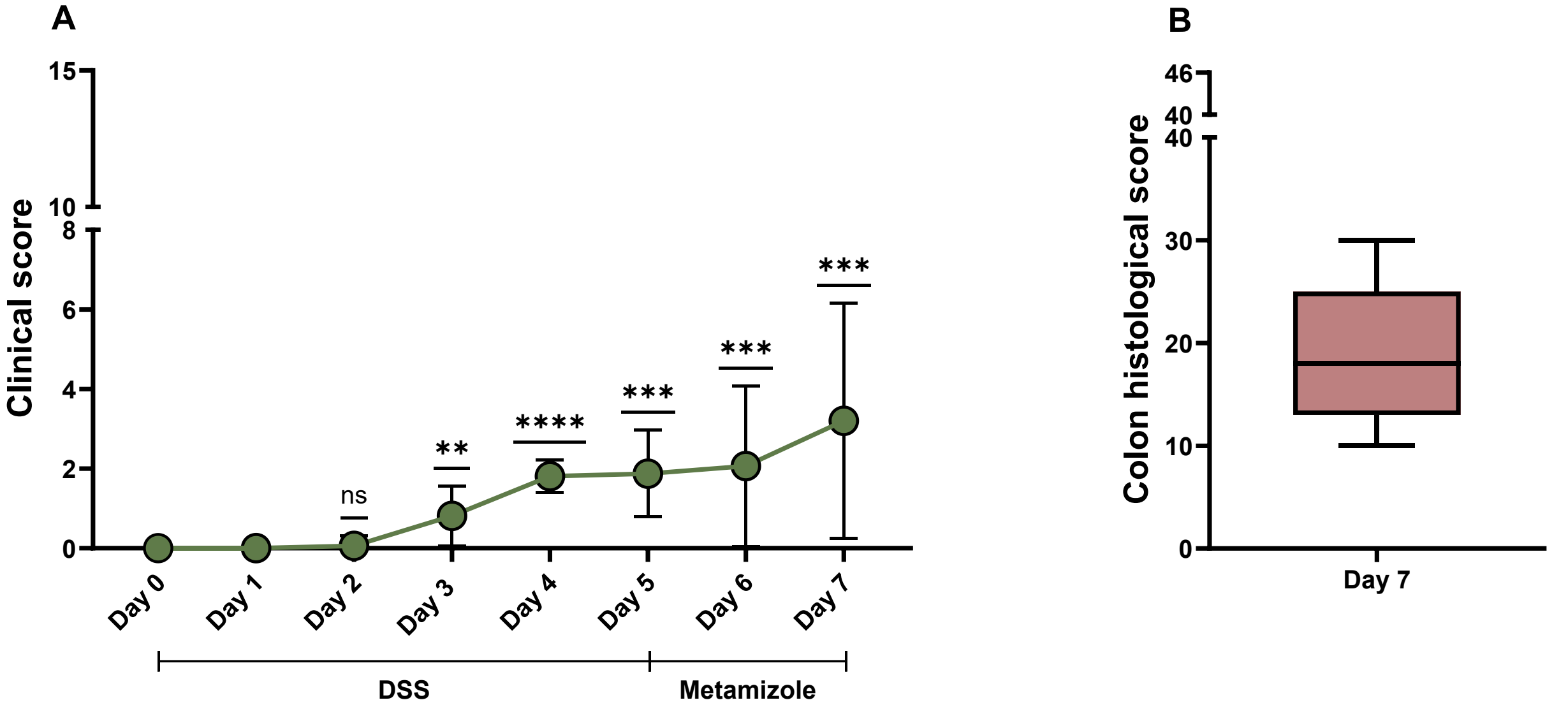

Supplementary Figure 3. Orbital tightening during DSS and metamizole administration. The groups were compared to each other using a Mann-Whitney-Test with the Holm-Šídák method. Within the groups, the change in the score was compared to Day 0 using the Wilcoxon signed-rank test to compare the score values with the baseline (hypothetical value of '0' (CG1 (§), CG2 (°), TG1 (#) and TG2 (*)). Only a significance of $p<0.0001$ within the in-group analysis is shown on the graph. The n= 16 in each group until day 7, n=14 on day 8, n=12 on day 9 and n=11 on day 10 in TG1; n=12 on day 8, n=10 on day 9 and n=10 on day 10 in TG2. DSS administration: d0-d5 in TG1 and TG2; Metamizole administration: d5-d10 in CG2 and TG2; The scores of the animals that were euthanized due to reaching the humane endpoint before the end of the experiment (TG1 and TG2 Days 9 and 7) are displayed with different colours on the graph. Values are plotted as Median with 95% CI. P was considered significant at ≤ 0.05 with * as $p\leq 0.05$; ** as $p\leq 0.01$; *** as $p\leq 0.001$; **** as $p\leq 0.0001$.

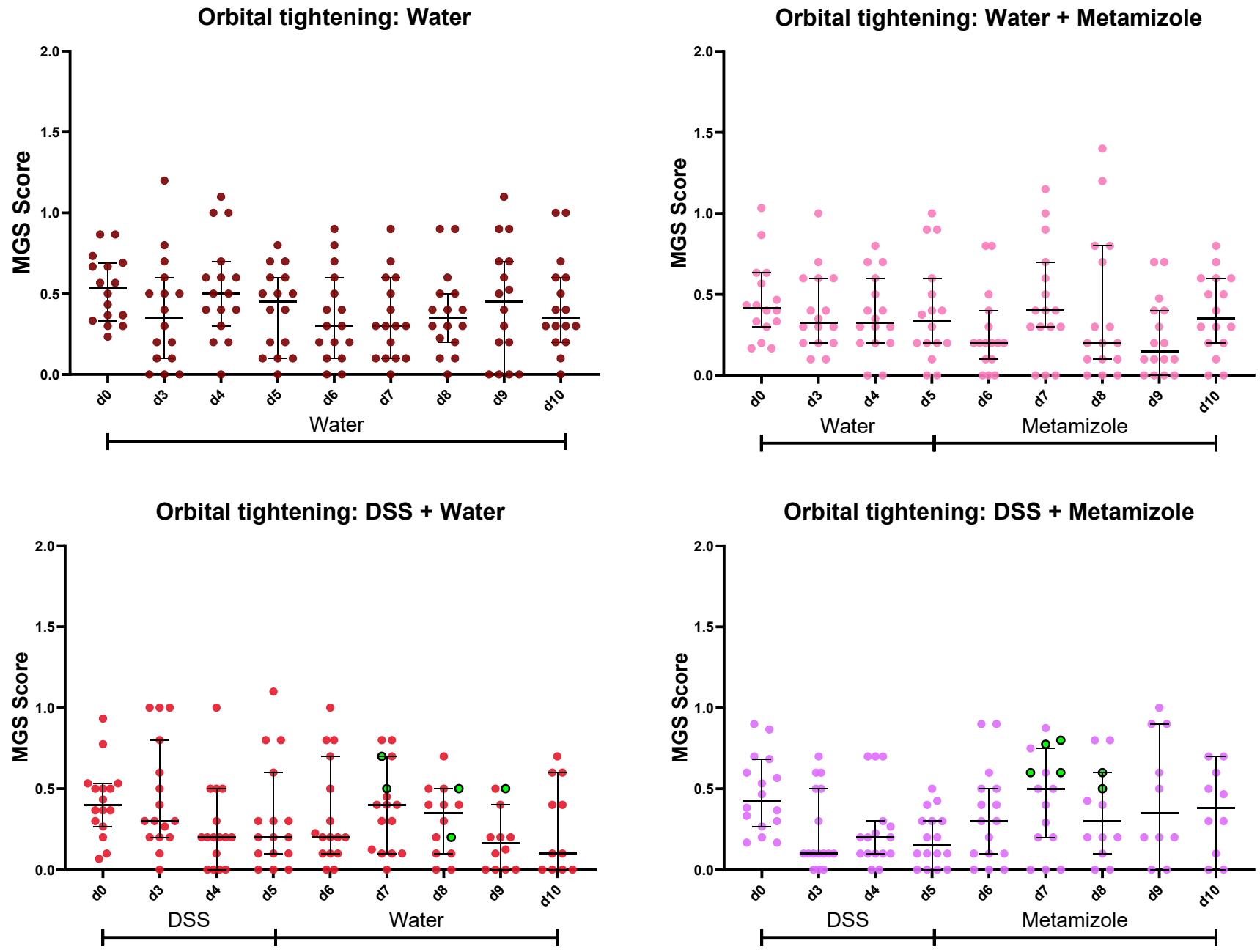

Supplementary Figure 4. Ear position during DSS and metamizole administration. The groups were compared to each other using a Mann-Whitney-Test with the Holm-Šídák method. Within the groups, the change in the score was compared to Day 0 using the Wilcoxon signed-rank test to compare the score values with the baseline (hypothetical value of '0' (CG1 (§), CG2 (°), TG1 (#) and TG2 (*)). Only a significance of $p<0.0001$ within the in-group analysis is shown on the graph. The n= 16 in each group until day 7, n=14 on day 8, n=12 on day 9 and n=11 on day 10 in TG1; n=12 on day 8, n=10 on day 9 and n=10 on day 10 in TG2. DSS administration: d0-d5 in TG1 and TG2; Metamizole administration: d5-d10 in CG2 and TG2; The scores of the animals that were euthanized due to reaching the humane endpoint before the end of the experiment (TG1 and TG2 Days 9 and 7) are displayed with different colours on the graph. Values are plotted as Median with 95% CI. P was considered significant at ≤ 0.05 with * as $p\leq 0.05$; ** as $p\leq 0.01$; *** as $p\leq 0.001$; **** as $p\leq 0.0001$.

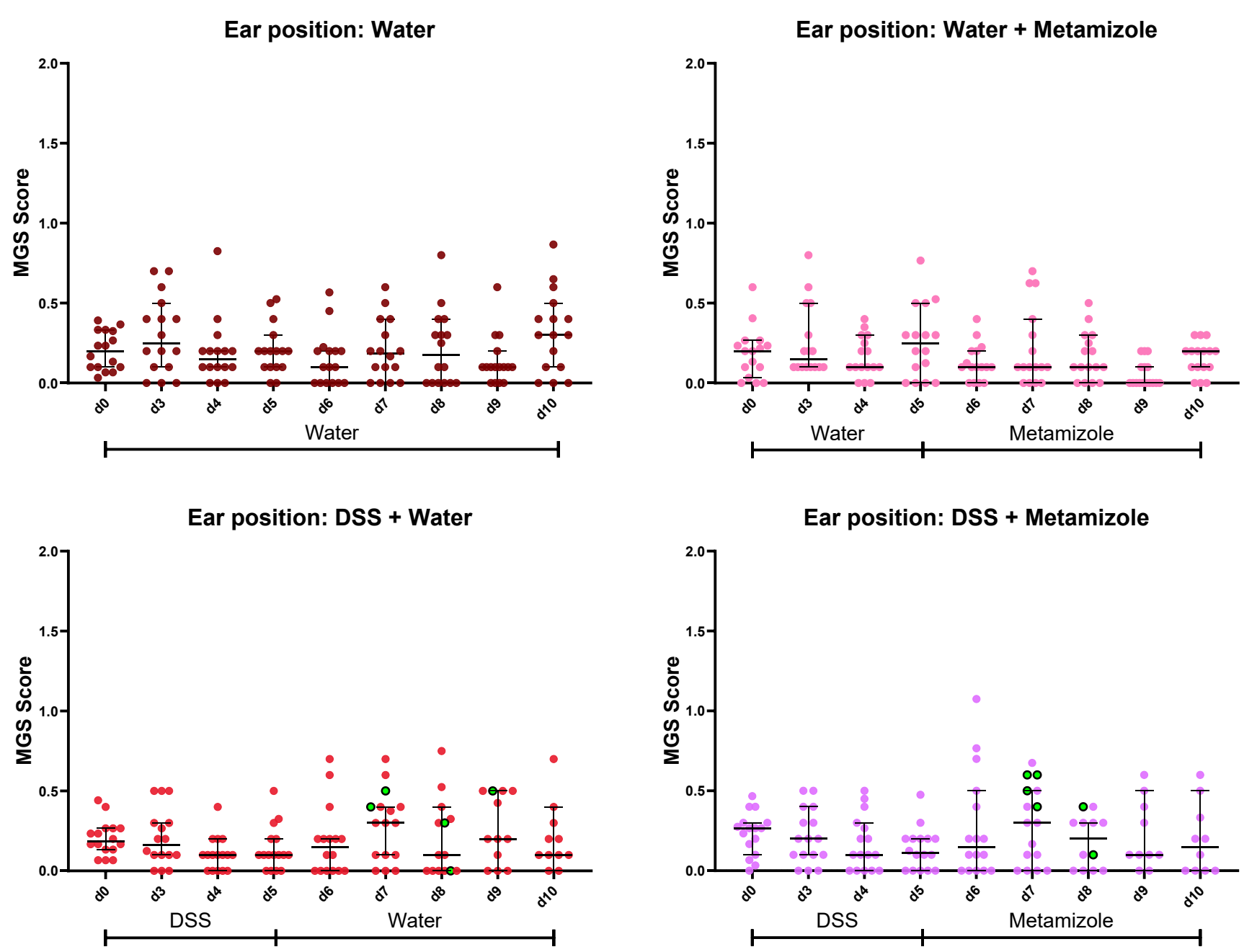

Supplementary Figure 5. Nose bulge during DSS and metamizole administration.The groups were compared to each other using a Mann-Whitney-Test with the Holm-Šídák method. Within the groups, the change in the score was compared to Day 0 using the Wilcoxon signed-rank test to compare the score values with the baseline (hypothetical value of ‘0’ (CG1 (§), CG2 (°) TG1 (#) and TG2 (*)). Only a significance of $p<0.0001$ within the in-group analysis is shown on the graph. The n= 16 in each group until day 7, n=14 on day 8, n=12 on day 9 and n=11 on day 10 in TG1; n=12 on day 8, n=10 on day 9 and n=10 on day 10 in TG2. DSS administration: d0-d5 in TG1 and TG2; Metamizole administration: d5-d10 in CG2 and TG2; The scores of the animals that were euthanized due to reaching the humane endpoint before the end of the experiment (TG1 and TG2 Days 9 and 7) are displayed with different colours on the graph. Values are plotted as Median with 95% CI. P was considered significant at ≤ 0.05 with * as p≤ 0.05; ** as p≤ 0.01; *** as p≤ 0.001; **** as p≤ 0.0001.

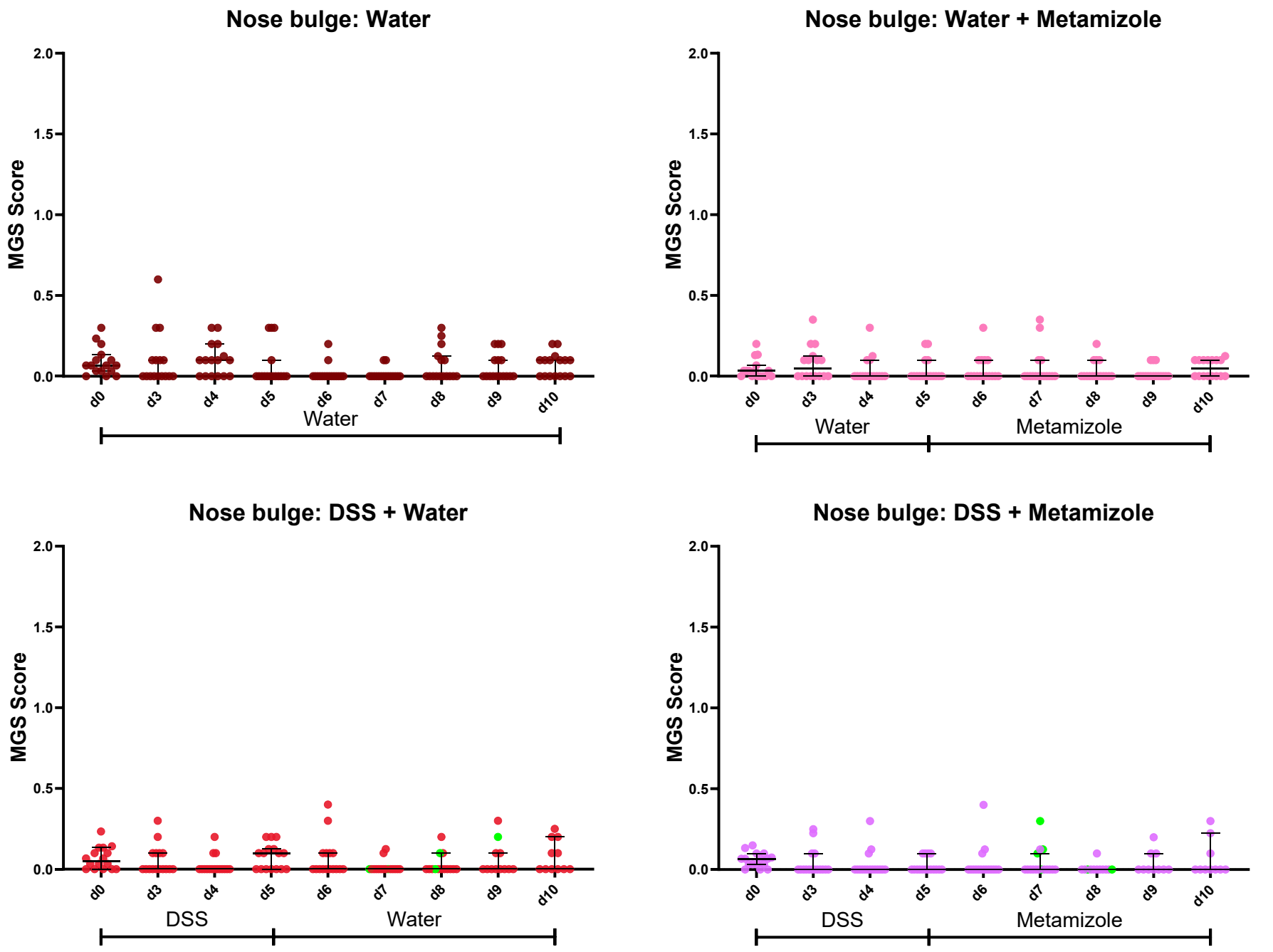

Supplementary Figure 6. Whisker change during DSS and metamizole administration. The groups were compared to each other using a Mann-Whitney-Test with the Holm-Šídák method. Within the groups, the change in the score was compared to Day 0 using the Wilcoxon signed-rank test to compare the score values with the baseline (hypothetical value of ‘0’ (CG1 (§), CG2 (°), TG1 (#) and TG2 (*)). Only a significance of $p<0.0001$ within the in-group analysis is shown on the graph. The n= 16 in each group until day 7, n=14 on day 8, n=12 on day 9 and n=11 on day 10 in TG1; n=12 on day 8, n=10 on day 9 and n=10 on day 10 in TG2. DSS administration: d0-d5 in TG1 and TG2; Metamizole administration: d5-d10 in CG2 and TG2; The scores of the animals that were euthanized due to reaching the humane endpoint before the end of the experiment (TG1 and TG2 Days 9 and 7) are displayed with different colours on the graph. Values are plotted as Median with 95% CI. P was considered significant at ≤ 0.05 with * as p≤ 0.05; ** as p≤ 0.01; *** as p≤ 0.001; **** as p≤ 0.0001.

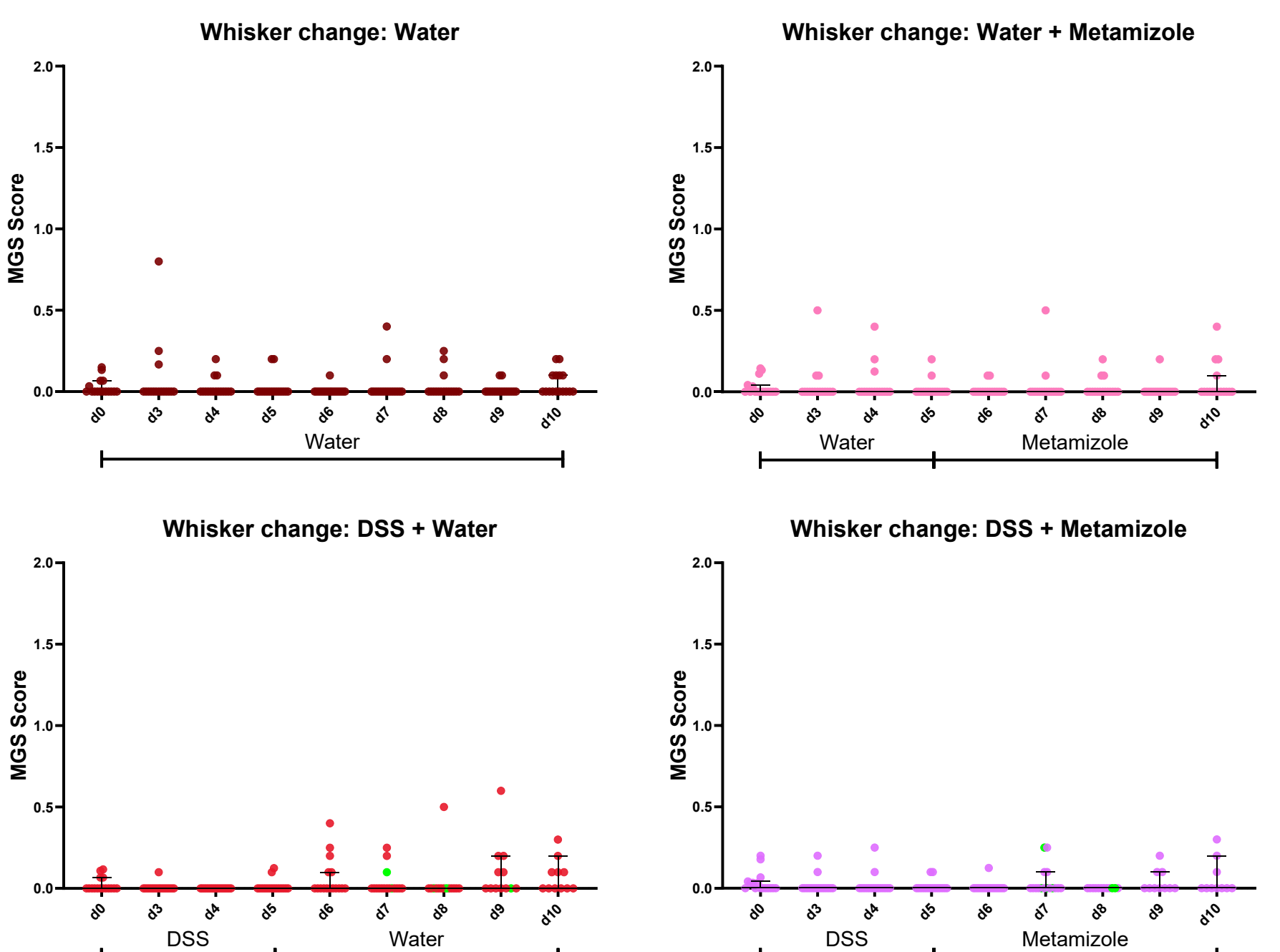

Supplementary Figure 7. Drinking water intake of the groups during DSS and metamizole administration. The intake from days 1- 7 were compared to the baseline using a RM one way ANOVA (p value: 0.0036; F (1.807, 5.421) = 19.41 followed by a Dunnett's multiple comparison test (p =<0.05) (n=16 from day 0 to day 6; n= 15 on day 7). P was considered significant at ≤ 0.05 with * as p≤ 0.05; ** as p≤ 0.01; *** as p≤ 0.001; **** as p≤ 0.0001. Blood sample collection indicated by the blue arrow.

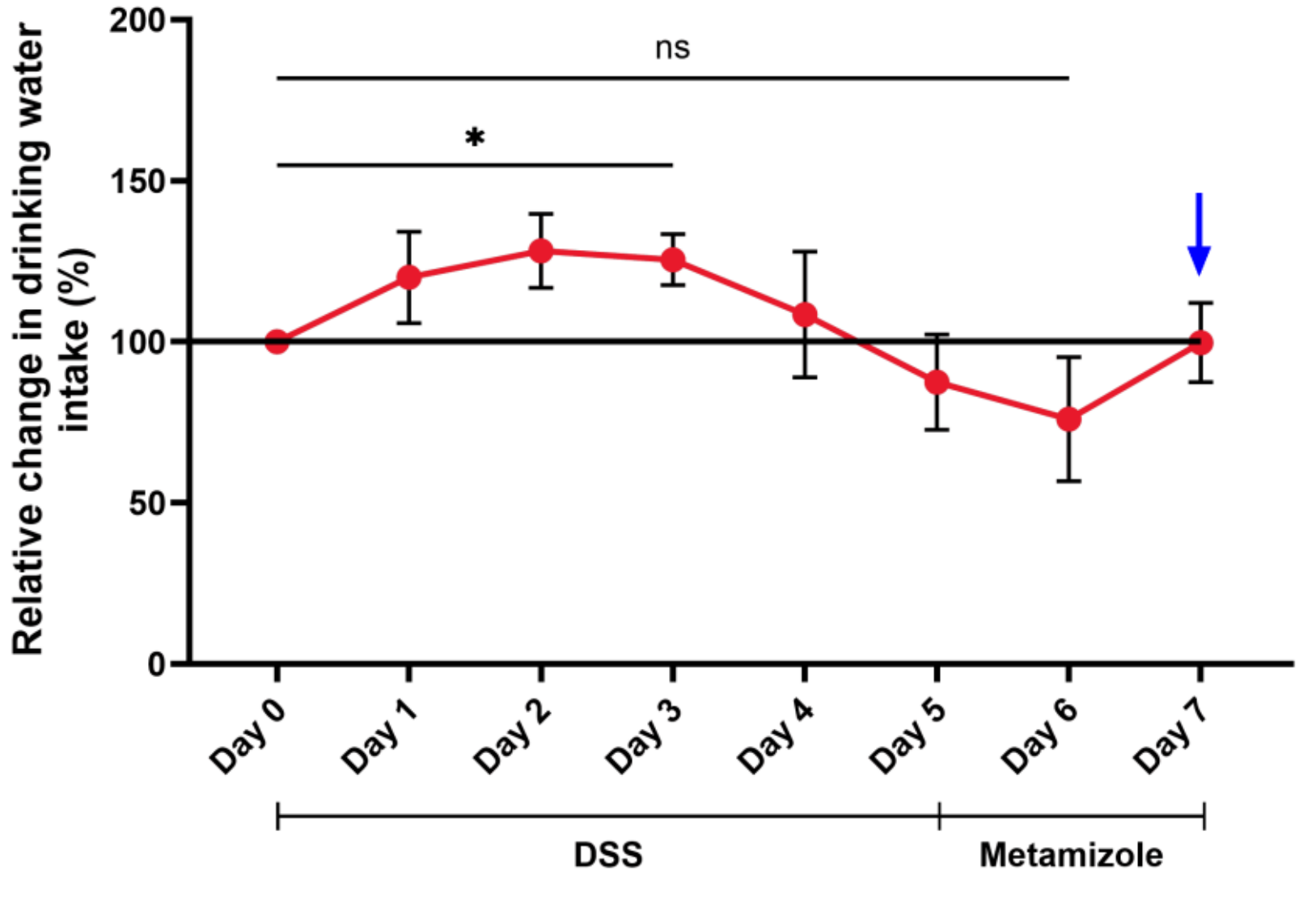

Supplementary Figure 8. Metamizole dosage during self-administration via drinking water. Metamizole dosage during self-administration after DSS-administration was compared to the target dose (200mg/kg/24h). The groups Water + Metamizole (CG2) vs. DSS + Metamizole (TG2) (°-°°°); were compared to each other using a Mixed-effects model (REML), Fixed effects (type III) followed by a Tukey's multiple comparison test (grey symbols). Within the groups, the intake was compared to the target dose (200mg/kg/24h) using a RM one-way Anova with a Dunnett's multiple comparison test CG2 (°) and a mixed-effects model (REML), Fixed effects (type III) followed by a Dunnett's multiple comparison test (TG2 (*)). None of the groups showed a significance of p<0.0001 in the in-group analysis. The n=4 in both groups till day 7, after which n= 3 in TG2. P was considered significant at ≤ 0.05 with * as p≤ 0.05; ** as p≤ 0.01; *** as p≤ 0.001; **** as p≤ 0.0001.

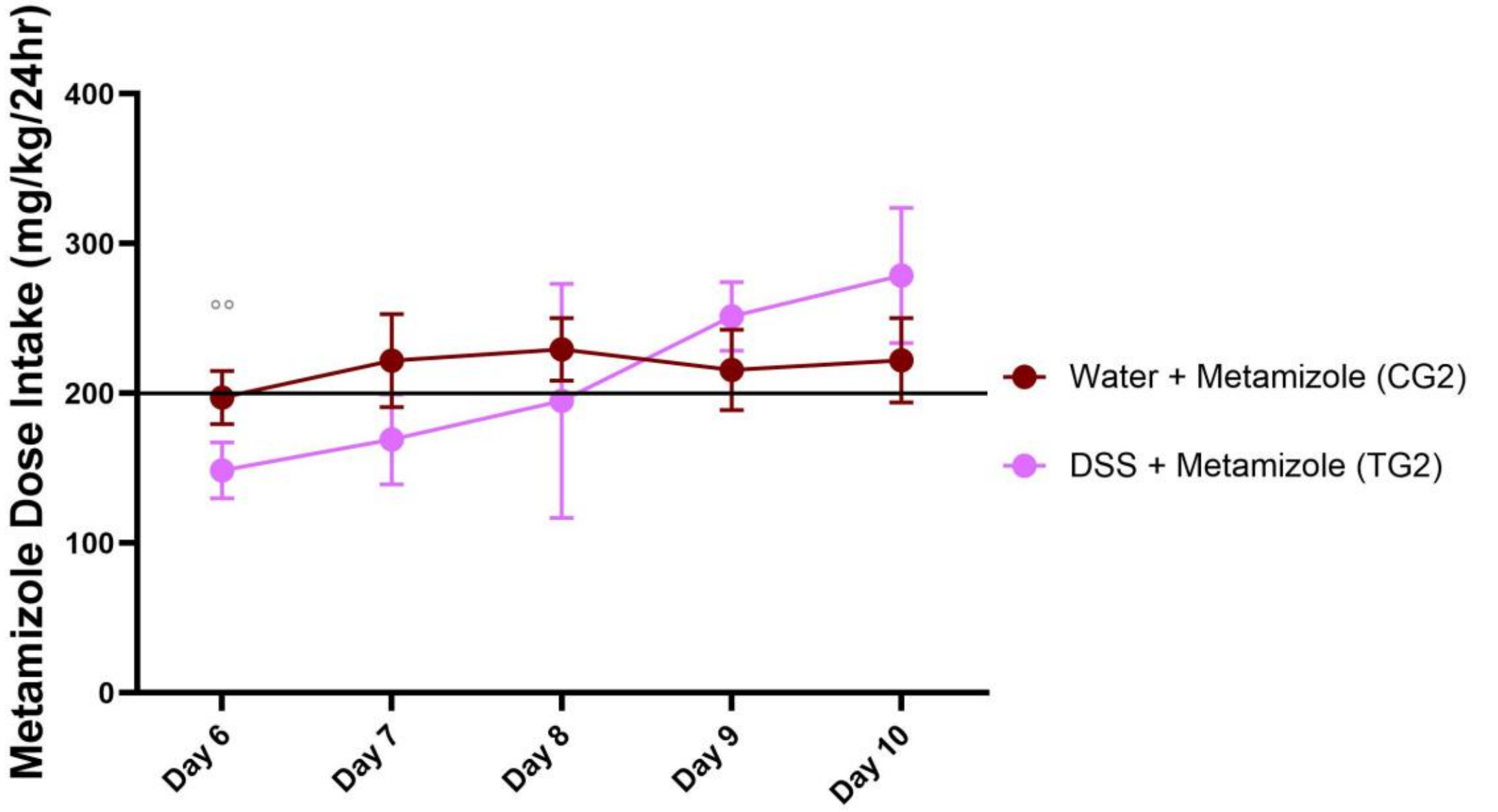

Supplement: Supplementary file 2 [file DataSheet2.pdf]
